# Advanced bone-targeted nanomaterials for the systemic treatment of osteoporosis

**DOI:** 10.1093/rb/rbag065

**Published:** 2026-04-01

**Authors:** Wenwen Fan, Pinzhuo Wu, Changsheng Liu, Xi Chen

**Affiliations:** Key Laboratory for Ultrafine Materials of Ministry of Education, Frontiers Science Center for Materiobiology and Dynamic Chemistry, Engineering Research Center for Biomedical Materials of Ministry of Education, School of Materials Science and Engineering, East China University of Science and Technology, Shanghai 200237, China; Key Laboratory for Ultrafine Materials of Ministry of Education, Frontiers Science Center for Materiobiology and Dynamic Chemistry, Engineering Research Center for Biomedical Materials of Ministry of Education, School of Materials Science and Engineering, East China University of Science and Technology, Shanghai 200237, China; Key Laboratory for Ultrafine Materials of Ministry of Education, Frontiers Science Center for Materiobiology and Dynamic Chemistry, Engineering Research Center for Biomedical Materials of Ministry of Education, School of Materials Science and Engineering, East China University of Science and Technology, Shanghai 200237, China; Key Laboratory for Ultrafine Materials of Ministry of Education, Frontiers Science Center for Materiobiology and Dynamic Chemistry, Engineering Research Center for Biomedical Materials of Ministry of Education, School of Materials Science and Engineering, East China University of Science and Technology, Shanghai 200237, China

**Keywords:** osteoporosis, nanomedicine, bone targeting, systemic administration, bone remodeling

## Abstract

Osteoporosis is a systemic skeletal disorder marked by reduced bone density and microstructural deterioration, leading to increased fragility and fracture risk. Current clinical treatments, mainly pharmacological, are limited by inadequate efficacy at vertebral sites, unidirectional inhibition of osteoclasts and systemic side effects. Nanotechnology offers a promising strategy to enhance drug delivery and bioavailability. Bone-targeted nanomaterial-based systems employing ligands such as bisphosphonates or peptides enable selective transport to bone tissue. Nanocarriers, including hydroxyapatite-based ones, can actively target osteoblasts or osteoclasts and support sustained release. This review synthesizes cellular-level insights into osteoporosis pathogenesis, critiques current therapeutic limitations and highlights advances in targeted nanomedicines. Specifically, it focuses on bone-targeting polymeric nanoparticle systems, which promote bone repair through multiple mechanisms: targeted drug delivery, modulation of the bone microenvironment, dual regulation of osteoblasts and osteoclasts, stimulation of bioactive signals from internal organs and restoration of mitochondrial homeostasis. By enhancing bone metabolism, these nanotherapeutic strategies present transformative potential for osteoporosis treatment and offer innovative directions for developing advanced regenerative biomaterials.

## Graphical abstract

**Graphical Abstract:**
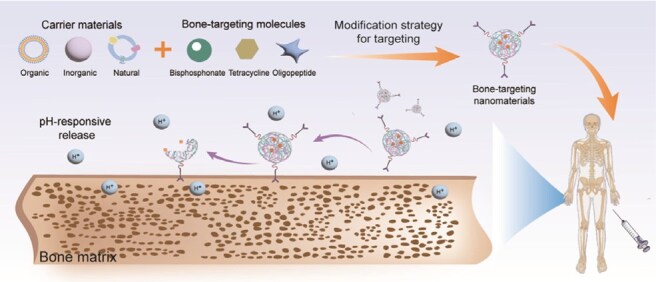

## Introduction of osteoporosis

Osteoporosis (OP, Supplementary Table S1 shows a list of abbreviations) is a systemic metabolic bone disorder characterized by reduced bone mass, deterioration of bone microarchitecture and an increased risk of fragility fractures [1, 2]. Key manifestations include decreased bone mineral density (BMD), disruption of bone tissue microstructure and heightened bone fragility [3]. As one of the most prevalent diseases worldwide, OP is associated with high rates of morbidity, disability and mortality, as well as substantial healthcare expenditures [4]. According to the World Health Organization, OP is diagnostically defined as a BMD value of 2.5 standard deviations or more below the mean for young healthy adults [5]. The disease significantly impairs functional independence and contributes to elevated morbidity and mortality among affected individuals [6]. Its pathogenesis is multifactorial, involving genetic, hormonal and environmental factors that collectively influence peak bone mass attainment, postmenopausal bone loss and age-related bone decline [7, 8].

From an etiological standpoint, OP is categorized into three types: primary, secondary and idiopathic. Primary OP constitutes the majority of cases and is predominantly attributed to postmenopausal estrogen deficiency (postmenopausal osteoporosis, PMOP) [9] and age-related skeletal deterioration (senile osteoporosis, SOP) [10, 11]. It is estimated to affect 50% of women and 25% of men over the age of 50, and OP exhibits a notably high prevalence among postmenopausal women [12]. In China, the prevalence of OP among adults aged 40 and above is 5.0% in men and 20.6% in women [13]. Epidemiological data show that the number of OP patients in China has reached 84 million, a figure anticipated to increase amid the country’s rapidly aging population [14]. In light of its growing prevalence and the associated high medical costs, OP has become a pressing public health challenge both in China and worldwide.

## Cellular mechanisms of osteoporosis

**Figure 1:**
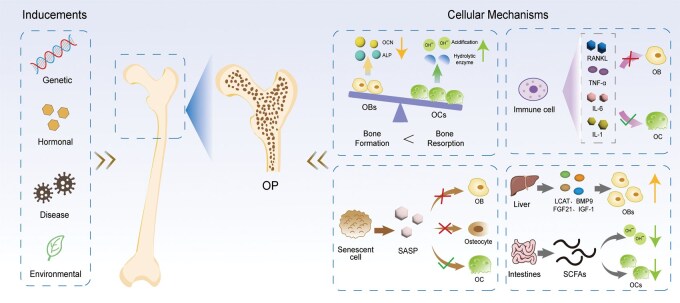
Pathogenesis of OP: Including common inducements and cellular mechanisms of action.

The pathogenesis of OP is fundamentally characterized by a disruption of homeostasis within the bone microenvironment. The bone microenvironment specifically denotes the dynamic local ecosystem within bone tissue where cells such as osteoblasts (OBs), osteoclasts (OCs) and osteocytes directly reside and perform their functions. In fact, this microenvironment encompasses not only bone cells but also immune cells (including macrophages and other cytokine-secreting immune cells) alongside mechanical stimuli, all of which collectively participate in regulating the bone remodeling process (Figure 1).

### Molecular mechanisms of osteoclast-mediated bone resorption and osteoblast-mediated bone formation

OCs originate from the hematopoietic stem cell (HSC) lineage, and their differentiation and maturation are consequently regulated by a diverse array of systemic cytokines and signaling molecules [15]. For instance, during OC differentiation, bone marrow-derived macrophages accelerate their differentiation in response to stimulation by the receptor activator of nuclear factor κB ligand (RANKL) [16]. OCs define the resorptive compartment by forming an actin-rich sealing zone, which confines the bone resorption activity to a targeted area [17, 18]. This process initiates with the firm attachment of OCs to the bone matrix, a critical preliminary step for resorption to occur. OCs then undergo polarization, leading to the formation of specialized subcellular structures such as the ruffled border and the actin ring. These structures are essential for enhancing the efficiency and localized activity of bone resorption [19, 20]. Subsequently, numerous endosomal vesicles fuse with the plasma membrane adjacent to the bone surface, forming a ruffled border within the sealed zone [21]. This highly specialized membrane structure serves as the primary site for bone resorption. Within this isolated compartment, OCs secrete a range of acids and hydrolytic enzymes [22]—including collagenase and other proteases—that degrade the organic components of the bone matrix, such as collagen [23–25]. Consequently, inorganic minerals, including calcium and phosphorus, are released from the bone matrix into the extracellular fluid [26–28].

OBs are pivotal cells in bone formation, responsible for synthesizing and secreting the bone matrix that facilitates osteogenesis and bone repair [29]. OBs are derived from mesenchymal stem cells (MSCs), primarily through the differentiation of bone marrow stromal cells [30, 31]. OBs facilitate bone formation through the secretion of various proteins that guide the deposition and organization of the bone matrix [12]. Key secreted factors include alkaline phosphatase (ALP) [32], osteocalcin (OCN) [33, 34], fibronectin [35] and osteopontin (OPN) [36], which serve as essential components of bone tissue and play crucial roles in promoting osteogenesis and bone repair. The matrix produced by OBs is rich in mineral components, particularly calcium and phosphorus. The deposition and mineralization of these minerals constitute an indispensable phase in bone formation and regeneration processes [37–39]. Bone mineralization occurs through two distinct stages: primary mineralization and secondary mineralization [40–43]. OBs initiate primary mineralization through the secretion of matrix vesicles, which serve as nucleation sites for initial mineral deposition. This is followed by a progressive increase in BMD during the secondary mineralization phase [44, 45]. ALP secreted by OBs can promote the deposition and mineralization of calcium ions in the bone matrix, and this process is a critical component of bone formation and repair [46–49].

Therefore, bidirectional regulation of osteogenesis and osteoclastogenesis—simultaneously activating OBs’ bone formation function and inhibiting OCs’ bone resorption function within the same intervention system—proves more effective in OP treatment strategies. Liang *et al*. [9] reported a CZA nanoparticle (NP) formed by loading the natural drug curcumin onto an acid-responsive zeolitic imidazolate framework-8 carrier and modifying it with the bone-targeting ligand aspartic acid octapeptide. Curcumin exerts its effects through a dual mechanism: it activates the osteogenic Wnt/β-catenin signaling pathway, promoting OB differentiation and bone matrix mineralization while upregulating osteogenic markers such as OCN and ALP, while simultaneously inhibiting RANKL-induced OC differentiation by downregulating OC markers such as CTSK and MMP9. This bidirectional synergistic effect significantly improves bone mass loss and microstructural deterioration associated with OP. Additionally, researchers including Chu *et al*. [50] reported a lanthanum-doped layered double hydroxide nano-hybrid scaffold. This scaffold activates the Wnt/β-catenin signaling pathway on the osteoblastic side via La^3+^, upregulating osteogenic genes, while blocking the RANKL-induced NF-κB pathway on the osteoclastic side to suppress osteoclastogenic genes. This dual action achieves bidirectional regulation of both osteogenesis and osteoclastogenesis. In molecular design, such technologies select or construct active components (e.g. curcumin, La^3+^) capable of simultaneously targeting the core yet opposing pathways “Wnt/β-catenin” and “RANKL/RANK/NF-κB.” Utilizing nanocarriers, they achieve lesion targeting (e.g. bone-targeting ligands) and microenvironment responsiveness (e.g. pH-responsive degradation) to ensure precise delivery of therapeutic payloads to active bone remodeling sites. This approach integrates the functions of delivery vehicles, physical scaffolds and active therapeutic units into a single system.

### Molecular mechanisms of immune cell regulation in bone remodeling

The immune system is intricately involved in skeletal physiology and pathology, with growing evidence suggesting that both innate and adaptive immune cells contribute to OP. Immune cells respond to various bodily conditions and trigger inflammation, with inflammatory diseases frequently associated with OP: inflammatory mediators such as reactive oxygen species (ROS) and pro-inflammatory cytokines and chemokines directly or indirectly act on bone cells and play a role in the pathogenesis of OP [51]. T lymphocytes (T cells) exacerbate PMOP by secreting specific cytokines that promote osteoclastogenesis and induce OB apoptosis. Under inflammatory conditions, activated B lymphocytes (B cells) enhance OC formation by increasing expression of RANKL. Neutrophils also enhance osteoclastogenesis, while mast cells release granules rich in OC-mediating substances such as interleukin (IL)-6 and tumor necrosis factor α (TNF-α) [52]. Beyond being producers of proinflammatory factors, macrophages can also serve as precursors for OCs. Depending on the microenvironment, macrophages can undergo polarization between M1 and M2 phenotypes. M1 represents the classically activated macrophage (inflammatory phenotype), and M2 represents the alternatively activated macrophage (repair phenotype). Macrophage polarization drives bone remodeling activities, proinflammatory cytokines like TNF-α and IL-6 stimulate M1 polarization, while anti-inflammatory cytokines such as IL-4 and IL-13 promote M2 polarization [53]. The heterogeneity and plasticity of macrophages make them key players in skeletal homeostasis.

Studies indicate that diminished mitochondrial oxidative phosphorylation (OXPHOS) function and disrupted histone/DNA modifications jointly contribute to bone loss [54]. Therefore, using mesoporous silica as a carrier and 4-octyl itaconate (OI) as an active molecule with dual immunometabolic and epigenetic regulatory activities, the surface was modified with a cerium ion-tannic acid (TA) network to construct MOCT NPs. This design enables sustained release of OI while leveraging cerium ions’ antioxidant capacity. Upon entering M1 macrophages, OI blocks inflammatory aerobic glycolysis while competitively inhibiting TET2 demethylase activity. This induces hypermethylation of pro-inflammatory gene (IL-6, CD86) promoters and reduces H3K27ac activation-type histone modifications, epigenetically locking them in a transcriptionally silenced state. Second, the cerium ion-TA network constructed on the NP surface utilizes Ce^3+^/Ce^4+^ to efficiently clear excess ROS accumulated in the bone microenvironment. This protects the functional integrity of mitochondrial respiratory chain complexes, restores OXPHOS-dependent adenosine triphosphate (ATP) synthesis and NAD^+^ regeneration, and provides the essential metabolic flexibility for macrophage phenotype conversion from M1 to M2. This approach offers a novel OP strategy integrating immunology, metabolism and epigenetics, featuring precise targeting and multifunctional synergistic advantages.

### Cell senescence and mitochondrial homeostasis imbalance lead to bone loss

Senescence is a stable cellular state marked by irreversible cell cycle arrest, evasion of apoptosis and the secretion of a plethora of inflammatory cytokines, chemokines and proteases—a phenomenon collectively termed the senescence-associated secretory phenotype (SASP) [55]. Within bone tissue, both OB precursors and mature OBs are susceptible to senescence. These senescent OBs display a markedly reduced capacity for osteogenic differentiation and are consequently impaired in their ability to form new bone matrix [56]. More critically, through their SASP, they secrete factors—including IL-6, IL-1β and matrix metalloproteinases (MMPs). These signals not only further autocrine suppress the function of neighboring OBs but also paracrine stimulate osteoclastogenesis. Notably, several SASP components, such as RANKL and IL-6, are potent inductors of OC differentiation and activation. Consequently, this process results in elevated OC activity and accelerated bone resorption. The SASP fosters a state of chronic, low-grade inflammation within the bone microenvironment [57, 58]. This phenomenon, often referred to as “inflammaging”, is a critical driver of disrupted bone homeostasis and exacerbated bone loss [59, 60]. Furthermore, aged bone marrow mesenchymal stem cells (BMMSCs) undergo a shift in their differentiation potential, exhibiting a preferential commitment to the adipocytic lineage at the expense of osteoblastogenesis. This shift contributes to increased bone marrow adiposity, a recognized hallmark of osteoporotic bone. Therefore, cellular senescence promotes bone loss through a dual mechanism: suppressing bone formation and enhancing bone resorption.

Mitochondria, the primary cellular energy generators, are integral to cell function through the regulation of their homeostasis, which encompasses biogenesis, dynamics (fusion and fission), quality control via selective autophagy (mitophagy) and metabolic function [61]. This regulatory network is especially critical in highly energy-dependent cells such as stem cells [62]. The processes of OB differentiation and new bone formation are exceptionally energy-intensive, requiring substantial ATP provision. Mitochondrial dysfunction compromises OXPHOS, leading to reduced ATP output. This energy deficit fails to meet the high biosynthetic demands of OBs, including collagen production and matrix mineralization, thereby impairing bone formation [63, 64]. Dysfunctional mitochondria represent a major intracellular source of ROS. The excessive ROS production resulting from mitochondrial impairment accelerates oxidative stress, which in turn promotes OB apoptosis and enhances OC activation [65, 66].

Mitochondrial DNA mutations and the accumulation of ROS are potent inducers of cellular senescence. Dysfunctional mitochondria promote the SASP through the release of ROS and bioactive metabolites, while SASP factors further compromise OB function. Consequently, cellular senescence and mitochondrial dysfunction engage in a vicious, self-reinforcing cycle: senescent cells exhibit progressively deteriorating mitochondrial function, and in turn, mitochondrial-derived ROS drive further cellular senescence. Collectively, these processes lead to a suppression of osteogenic differentiation and an exacerbation of OC activity. This imbalance ultimately results in impaired bone formation, the deterioration of bone microarchitecture and a net loss of bone mass.

### Molecular mechanisms of multidimensional inter-organ communication in regulating bone homeostasis

In recent years, the academic community has increasingly recognized that bone remodeling is not solely driven by local mechanical stimuli or traditional calmodulin-dependent hormones, but rather represents a dynamic process involving continuous chemical signaling from multiple organs to the skeleton. Among these, the “liver-bone axis” is a bidirectional metabolic regulatory pathway proposed in recent years, referring to the physiological and pathological connections between the liver and bones mediated by various hormones, cytokines and signaling molecules. The liver promotes bone mass maintenance by secreting bone morphogenetic protein 9 (BMP9), fibroblast growth factor 21 (FGF21) and insulin-like growth factor 1 (IGF-1) to stimulate OB differentiation. Additionally, liver-secreted lecithin cholesterol acyltransferase (LCAT) enhances OB differentiation while inhibiting OC differentiation. Meanwhile bones exert feedback effects on hepatic metabolism, glucose-lipid homeostasis and fibrosis progression via factors including OCN, sclerostin and transforming growth factor (TGF)-β [67]. The “liver-bone axis” highlights the critical role of hepatic metabolic homeostasis in the pathogenesis of OP, offering novel potential directions for the prevention, diagnosis and treatment of bone-related diseases.

A normal intestinal barrier is crucial for promoting the absorption of vitamin D and calcium ions while preventing harmful substances from crossing the intestinal epithelium. Dysbiosis of the gut microbiota may induce OP by impairing intestinal barrier function in rats [68]. Furthermore, the gut microbiota may influence skeletal remodeling through microbiota-derived metabolites such as short-chain fatty acids (SCFAs). SCFAs lower intestinal pH, enhancing the solubility of calcium, magnesium and phosphorus, while simultaneously suppressing OC gene expression of NFATc1 and TRAF6, thereby reducing bone resorption [69]. During intestinal dysbiosis, translocated Gram-negative bacteria and their lipopolysaccharides (LPS) activate NF-κB inflammatory pathways in bone marrow and periosteal regions, upregulating TNF-α, IL-1 and IL-6 to promote OC differentiation, while probiotics can expand Tregs, increase IL-10 and TGF-β, inhibit Th17-mediated bone destruction and restore bone remodeling balance [70]. Excessive immune activation, manifesting as chronic inflammation, is a primary driver of pathological bone resorption and homeostatic disruption. In chronic inflammatory states—including ageing and autoimmune diseases—cytokines such as TNF-α, IL-1β and IL-6 potently stimulate osteoclastogenesis. Moreover, immune cells such as T cells and B cells directly regulate OC activity through the secretion of key mediators including RANKL.

A bidirectional regulatory relationship also exists between muscle and OP: reduced muscle mass accelerates bone loss, while decreased bone strength in turn promotes muscle atrophy. The two interact through mechanical forces, endocrine pathways, inflammation and molecular signaling. Clinically, the concurrent condition is often termed “sarcopenia-OP” [71]. From a biomechanical perspective, muscle force constitutes the primary mechanical component generating skeletal strain. Consequently, diminished muscle function and performance reduce skeletal loading, leading to worsened bone density. Additionally, muscle paralysis, atrophy or immobilization similarly contribute to bone loss and OP [72]. As an endocrine organ, muscle secretes multiple myokines, including irisin, myostatin, IGF-1 and FGF2. Irisin, stimulated by exercise in muscle fibers, promotes osteogenesis and inhibits osteoclastogenesis via the Wnt/β-catenin pathway. Myostatin, however, exerts negative regulation not only in muscle but also in bone, directly promoting OC recruitment and differentiation [73]. Bone-derived factors, including OCN and prostaglandin E2 (PGE2), also influence muscle function. Thus, maintaining or increasing muscle mass not only improves balance and reduces falls but also directly decreases bone resorption and promotes bone formation. Conversely, preventing and treating OP necessitates concurrent attention to muscle health.

## Clinical treatment of osteoporosis and application research of nanotechnology

### Traditional therapies for osteoporosis

OP is currently managed through a combination of pharmacological and nonpharmacological interventions [74]. Antiresorptive agents—such as estrogens, bisphosphonates, calcitonin and selective estrogen receptor modulators (SERMs)—constitute the most widely used pharmacological class for OP treatment [75–78]. Anabolic drugs, including fluorides, parathyroid hormone (PTH), androgens and vitamin K, are typically reserved for cases of severe OP [79, 80].

However, current pharmacological interventions for OP are not only limited in efficacy but are also associated with inevitable adverse effects. These treatments suffer from several drawbacks, including prolonged duration, high demands on patient adherence and inadequate local drug concentrations—all of which contribute to suboptimal therapeutic outcomes [81, 82]. Furthermore, long-term drug administration often leads to systemic side effects [36, 83]. For instance, antiresorptive agents such as bisphosphonates and inorganic pyrophosphate analogues act primarily by inhibiting farnesyl pyrophosphate synthase in OCs, leading to OC detachment from the bone surface, reduced resorptive activity and ultimately suppression of bone loss [84–86]. Nonetheless, bisphosphonates lack direct osteogenic effects, necessitating higher doses and extended treatment periods, which in turn increase the risk of adverse events such as gastrointestinal complications or atypical fractures [87, 88]. Most anabolic agents-including recombinant human PTH (represented by teriparatide) and PTH-related peptide (represented by abaloparatide) require invasive routes of administration such as injection, further complicating long-term therapy [89, 90]. However, this systemic mode of administration has not been demonstrated to significantly enhance the healing of systemic bone defects [91]. Moreover, patient tolerance to long-term use of these agents is often poor, with associated adverse effects including headache, nausea [92] and an increased risk of osteosarcoma [93]. Consequently, peptide-based therapies are generally reserved for short-term treatment in cases of severely low bone mass, with usage typically restricted to a maximum of 24 months [94]. Similarly, the direct administration of SERMs may induce side effects such as vasomotor disturbances and venous thromboembolism [95, 96]. In summary, while traditional antiresorptive and pro-resorptive drugs are effective, their long-term use is often accompanied by adverse reactions. They also suffer from inherent limitations such as low bioavailability, poor targeting and low patient compliance. The emergence of nanotechnology offers a new direction for overcoming these bottlenecks.

### Innovative advances in nanomaterials for osteoporosis treatment

Researchers are increasingly turning to nanotechnology-based strategies to develop novel therapeutic platforms. By designing drug delivery systems ranging in size from 1 to 1000 nanometers, these systems can enter the systemic circulation via routes such as intravenous injection. This enables controlled drug release and precise delivery, allowing intervention at the cellular and subcellular levels to address the root causes of bone metabolism. Surface modification with molecules exhibiting high bone affinity—such as alendronate (ALN) sodium and aspartic acid oligopeptides—NPs can achieve specific accumulation at bone lesion sites, significantly enhancing local drug concentrations while minimizing interference with nontarget organs like the heart, liver and kidneys. Research [97] demonstrates that precisely controlling the concentration of nano-hydroxyapatite (HAP) can “program” its osteogenic effects. Within cells, HAP NPs slowly dissolve, releasing calcium ions. For osteoporotic cells with disrupted calcium homeostasis, this supplemental external calcium source precisely “corrects” their functional defect. This activates osteogenic signaling pathways, selectively promoting bone repair in osteoporotic conditions while avoiding excessive stimulation of healthy bone. This approach overcomes limitations inherent in traditional materials. Additionally, Zhao *et al*. [98] designed and prepared a biomimetic mineralizing hydrogel termed CHAp-PAA. Fabricated through the supramolecular assembly of nano-HAP, sodium carbonate and polyacrylic acid, it is engineered to mimic the chemical composition and structural architecture of natural bone matrix.

Peptide-based nanostructures constitute self-assembling nanofibrous frameworks capable of complexing with nano-HAP/tricalcium phosphate composites. This forms a three-dimensional microenvironment characterized by high specific surface area and abundant cell recognition sites, significantly enhancing the adhesion, osteogenic differentiation and collagen/calcium phosphate matrix deposition of BMMSCs calcium matrix deposition. This nano-tissue engineering approach utilizing self-assembled peptide nanofibers (SAPNs) offers a breakthrough method for repairing critical bone defects, overcoming limitations in bone induction, angiogenesis and immunomodulation associated with traditional ceramic grafts [99].

Polymer-ceramic composite systems related to bone mineral composition refer to the integration of inorganic mineral phases (primarily HAP) from natural bone with organic polymer matrices. Through biomimetic design, these systems achieve synergistic optimization of mechanical properties, bioactivity and degradation behavior for bone defect repair and regeneration. Zhao *et al*. [100] employed hydrothermal synthesis to produce tea polyphenol-functionalized micro-/nano-HAP bioceramics (TP-nwHA), whose structure closely resembles the bone-like apatite architecture induced by HAP bioceramics *in vivo*. This enhances cell proliferation and adhesion while significantly influencing MSCs, promoting osteogenic differentiation and simultaneously inhibiting OC differentiation. It proposes a novel strategy for fabricating bone-mimetic structures with potential applications in disease models.

However, most current nanotechnology-based therapeutic strategies remain confined to preclinical research, hindered by a significant translation gap. Although *in vitro* and animal studies have yielded promising results, critical uncertainties remain concerning the long-term *in vivo* biodistribution, immunogenic potential and organ-specific toxicity of these nanomaterials, as well as their generalizability across diverse osteoporotic etiologies. Moreover, existing efforts have primarily emphasized proof-of-concept efficacy, with comparatively limited analysis of mechanistic depth, clinical feasibility and scalable manufacturing challenges. In summary, nanotechnology demonstrates significant advantages in OP treatment through intelligent response-controlled release, modulation of the immune microenvironment and delivery of novel therapeutic molecules (such as gene drugs). However, these advanced technologies face a fundamental challenge in practical application: ensuring therapeutic carriers can efficiently and specifically reach skeletal lesion sites. It is precisely to overcome the widespread limitations of NPs delivery within the body that “bone-targeting technology” has emerged as the core component for constructing highly efficient nanotherapeutic platforms.

## Current advances in innovative bone-targeted nanomaterials for osteoporosis

**Figure 2:**
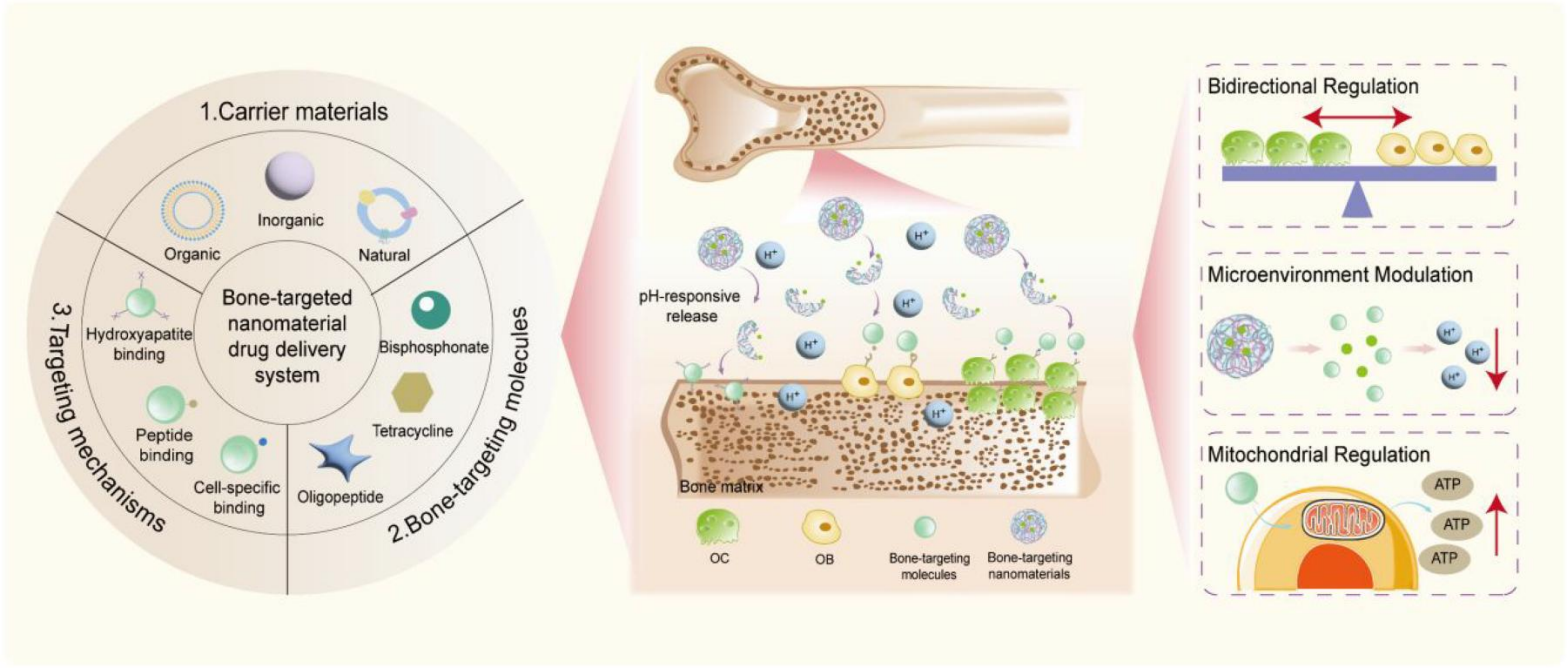
Current status of OP treatment and mechanism of action of the bone-targeted nanomaterial drug delivery system.

As previously mentioned, bioactive nanomaterials have emerged as highly promising alternative solutions for treating OP. Bioactive nanomaterials refer to materials that are at least one-dimensional in the nanoscale range and capable of controlled, beneficial interactions with biological environments (such as body fluids, cells and tissues), thereby guiding desirable biological responses. They can also function as stimulus-responsive carriers for the controlled release of chemical and bioactive agents, thereby enhancing therapeutic efficacy [101, 102]. NPs can be fabricated from a diverse range of materials, offering extensive flexibility for achieving tailored therapeutic functions. These include organic NPs such as liposomes [103, 104], and polymeric NPs [105] as well as inorganic varieties, including HAP and metallic NPs [106, 107] (Figure 2). For instance, in hyperthermia-based treatments aimed at disrupting OC activity in OP, iron oxide-based nanomaterials such as Fe_3_O_4_ NPs can be utilized. Their strong magnetic responsiveness enables remote modulation of OC function [108]. Conversely, for targeted delivery of genes or drugs, novel liposomal systems represent an effective nanomaterial platform for constructing precision delivery systems [109]. Thus, the selection of nanomaterials can be strategically aligned with specific therapeutic objectives in OP management. Furthermore, NPs can be co-loaded with multiple drugs and bioactive factors, enabling multifunctional therapeutic capabilities. Chitosan, one of the most widely used polymers in drug delivery, serves as a representative example [110]. A recently developed cartilage-mimetic scaffold incorporating icariin into a short-fiber-reinforced chitosan hydrogel not only enhances osteocyte proliferation but also exhibits anti-inflammatory effects and provides mechanical support. Additionally, this system allows for controlled release of therapeutic agents, exemplifying the potential of nanotechnology-based platforms as multifunctional biomaterials.

Researchers have increasingly turned to nanotechnology-driven strategies to develop novel therapeutic platforms. For example, targeted nanoscale drug delivery systems can improve treatment outcomes in OP by enhancing drug efficacy, minimizing adverse effects, reducing systemic toxicity and improving the pharmacokinetic profiles of therapeutic compounds [111]. Currently, various NP systems have been developed for OP treatment, operating primarily through two mechanistic pathways: inhibition of bone resorption or stimulation of bone formation [112, 113]. As previously indicated, systemically administered drugs often require high dosages to achieve therapeutic efficacy, which can exacerbate their toxicity profiles. NP-based drug delivery systems mitigate this issue by reducing the required drug dosage and minimizing associated adverse reactions [114]. For instance, a study developed echinacoside-loaded cross-linked NPs that effectively suppress oxidative stress, decrease ROS generation and inhibit OC formation—achieving significant therapeutic outcomes at lower concentrations while reducing drug-related toxicity compared to free drug administration [115]. Furthermore, certain free drugs may induce off-target adverse effects. Estrogen therapy, for example, is associated with extraskeletal risks such as breast cancer and uterine bleeding. By conjugating estradiol (E_2_) with NP carriers, targeted delivery to bone tissue can be achieved, thereby avoiding undesirable systemic effects, prolonging the dosing interval and enhancing patient quality of life [116]. Therefore, the advantage of NPs lies in their ability to be engineered through adjustments to their composition, structure or surface modifications, thereby enabling controlled drug release under specific conditions or at specific times [117]. For example, a class of NPs functionalized with cell-mimetic membranes, bone-targeting moieties and ROS-responsive linkers not only exhibits enhanced affinity for bone tissue but also contains thioketal (TK) bonds that cleave upon ROS exposure, resulting in the release of mitochondria-targeting peptides. This design enables precise spatiotemporal control over drug delivery [118]. Additionally, nitric oxide (NO), which promotes osteogenesis and has shown potential in reversing OP, can be released in a light-controllable manner. NPs capable of catalyzing NO generation from dinitrosyl iron complexes allow for precise and tunable release of NO, offering a promising strategy for regulating bone remodeling processes [119].

In recent years, a variety of more cutting-edge nanomaterial designs have emerged in this field. MicroRNA (miRNA)-based nanotherapeutic systems focus on precisely delivering specific miRNAs to diseased cells via nanomaterials to regulate gene expression and treat diseases. This technology aims to overcome the limitations of traditional drug delivery methods, achieving more efficient and precise treatment, and has thus, been applied to OP therapy. For instance, one study [120] developed triple-wristed junction 3WJ RNA NPs-targeting MSCs. Utilizing 3WJ RNA NPs as delivery carriers, the RNA triple-wristed junction structure serves as a core scaffold—stable and amenable to functionalization—enabling specific delivery of antimiRNA 138. Following uptake by MSCs, the NPs successfully suppressed intracellular miR-138 expression, significantly promoting MSC differentiation into OBs and enhancing the expression of osteogenesis-related genes, thereby achieving the goal of treating OP. This study proposes and validates a highly targeted, modular RNA nanotherapeutic platform. Its core advantage lies in integrating cell-specific targeting (aptamer), stable drug delivery (3WJ scaffold) and effective gene regulation tools (LNA-antimiR) within a single NP. This approach enhances therapeutic efficacy while potentially reducing side effects caused by off-target effects. Additionally, methods for cell membrane-coated NPs (CMNPs) have also proliferated rapidly, aiming to enhance the skeletal microenvironment and thereby improve the efficacy of antiresorptive therapies. The core concept involves achieving systemic administration via intravenous injection or similar routes, leveraging the “natural camouflage” and “homing” capabilities of cell membranes to deliver therapeutic agents precisely and efficiently to skeletal lesions while minimizing side effects on other tissues throughout the body. Zhang *et al*. [118] encapsulated polymeric NPs within bone marrow-derived mesenchymal stem cell membranes, incorporating bone-targeting molecules (ALN). This approach achieved immune evasion, bone targeting and intelligent drug release at the lesion site, with no significant systemic side effects. Additionally, nanocarrier technology for advanced gene regulation methods aims to address the challenges faced by gene therapies (such as DNA, siRNA, miRNA, etc.) in systemic applications, namely susceptibility to degradation and difficulty in efficiently reaching target cells. By designing specific nanocarriers, key genes regulating bone metabolism and small interfering RNA can be efficiently and specifically delivered to diseased cells throughout the skeletal system, thereby correcting bone metabolic imbalances at the genetic level. Li *et al*. [121] developed a novel nucleic acid delivery system based on degradable cationic polymers and ALN groups to deliver therapeutic miRNA to bone tissue. This approach suppressed tumor and alleviated bone resorption in bone metastasis models, with the study indicating its potential extension to OP treatment.

Despite promising prospects, most bone-targeting nanomedicine systems remain largely in the early preclinical stages. Their translation into clinical practice is hindered by several formidable challenges that demand urgent and critical analysis. First, existing models have inherent limitations. The current compelling evidence predominantly comes from cell lines and rodent models, which cannot fully recapitulate the complex, multifactorial pathophysiology of human OP, including its chronic progression and systemic comorbidities. Second, targeting efficiency and specificity are unresolved. While most strategies exploit affinity for bone mineral (HAP) to achieve tissue-level accumulation, they lack the precision to distinguish between key cellular players within the bone microenvironment—such as senescent MSCs, overactive OCs or dysfunctional OBs. This limited cellular specificity may curtail therapeutic efficacy and raises concerns about off-target effects. Finally, long-term biosafety constitutes a major barrier. The intricate interactions between nanomaterials and biological systems are poorly understood. The ubiquitous sequestration of NPs by the mononuclear phagocytic system in the liver and spleen poses risks of chronic inflammation, fibrosis and disruption of systemic endocrine homeostasis.

### Development of a bone-targeted drug administration system

Therapeutics can be administered via adhesion to the carrier surface or encapsulation within the carrier matrix, followed by controlled release at the target site. Bone-targeted carrier materials (Table 1) are engineered substances designed to load therapeutic agents and deliver them to specific bone tissues or cellular structures through tailored functionalization [122]. The selection is extremely broad, with carrier materials that can be flexibly chosen based on drug properties (water-soluble/lipid-soluble) and therapeutic requirements. These carriers significantly influence drug stability, release kinetics, *in vivo* biodistribution and overall therapeutic efficacy [123, 124]. As nanocarriers for targeted drug delivery, they are designed based on several strategic considerations: leveraging intrinsic physicochemical properties-such as particle size, surface charge and morphology—to enhance tissue penetration and interaction, and enabling active targeting through surface modification with specific ligands or the construction of biomimetic nanostructures capable of recognizing and binding to particular tissues or cell types [22]. Conventional drug carriers commonly employed in such systems include organic nanomaterials, inorganic nanomaterials and cell-derived biomimetic nanomaterials [125, 126].

**Table 1:** Typical bone-targeting carrier materials.

Classification	Biomaterials	Advantages	Disadvantages	Reference
Organic nanomaterials	Liposomes	Improve the chemical stability of materials Increase the relative bioavailability of the material Realize sustained and controlled drug release	The metabolic clearance pathway remains unclear Induce potential toxicity of metabolic byproducts Complicate the preparation process	[133, 134]
	Polymeric NPs	Enhance drug solubility Prolong the drug release cycle Achieve controlled release and targeted drug delivery	Reduce drug loading capacity and encapsulation efficiency Compromise storage stability Induce potential toxicity	[135–137]
	Micelles	Enhance bone-targeting bioactivity Enhance the bioavailability of hydrophobic drugs Achieve sustained and controlled release	Result in a complex synthesis process Reduce drug loading capacity and stability Compromise biological safety	[138, 139]
Inorganic nanomaterials	Mesoporous silica	Increases the drug loading capacity Enhances storage stability Achieves stimuli-responsive controlled release	Reduces biodegradability Leads to potential cytotoxicity Reduces the rate of biodegradation	[140, 141]
	Iron oxide NPs	Achieve multifunctionality and synergistic therapy Increase the drug loading capacity Promote osteogenic differentiation	Induce dose-dependent cytotoxicity Induce inflammatory reactions and prolonged retention Restrict the application of magnetic fields	[142]
	Metal NPs	Possess excellent biocompatibility Achieve surface functionalization Achieve stimuli-responsive drug release	Result in a complex synthesis process Cause potential immunogenicity The metabolic clearance pathway remains unclear	[143–145]
Natural biological nanomaterials	Extracellular vesicles (EVs)	Enhance biocompatibility Reduce immunogenicity Enhance osteogenic activity	Reduce purity and yield Reduce drug loading efficiency The metabolic clearance pathway remains unclear	[2, 146]

Bone-targeting molecules are a class of functional molecules or ligands capable of specifically recognizing, binding to and accumulating within bone tissue (particularly the bone mineral matrix; Table 2) enable the specific delivery of drugs and other therapeutic agents to bone tissue, playing a critical role in the diagnosis and treatment of skeletal disorders. By utilizing these targeting ligands, drug concentrations can be significantly enhanced at pathological bone sites, thereby improving therapeutic efficacy, while simultaneously reducing off-target distribution and minimizing systemic side effects [127, 128]. Bone-targeted drug delivery systems offer the significant advantage of high targeting specificity. Through surface modification, nanomaterials can achieve precise delivery to bone tissue, thereby enhancing local drug concentration while reducing toxicity to nontarget tissues [129, 130]. Furthermore, nanomaterials improve drug bioavailability and decrease the rate of drug degradation and clearance *in vivo* [131]. These properties collectively mitigate systemic side effects, enhance treatment safety and ultimately lead to substantially improved therapeutic outcomes [132]. The design of effective bone-targeting drug delivery systems must fulfill several critical criteria. First, the material surface requires functionalization with specific bone-targeting moieties, such as bisphosphonates or tetracyclines, to achieve selective binding to bone mineral components, primarily HAP. Physicochemical properties, including particle size and surface charge, must be precisely regulated; an optimal size range of 50–500 nm is essential to evade rapid renal clearance and facilitate tissue penetration. Furthermore, the carrier material itself must demonstrate excellent biocompatibility and be capable of undergoing controlled degradation or metabolism after fulfilling its delivery function, thereby avoiding long-term *in vivo* accumulation. High drug encapsulation efficiency is necessary to minimize premature leakage during systemic circulation. Equally important is the capacity for stimuli-responsive drug release within the distinctive bone microenvironment—triggered by local conditions such as elevated glutathione concentration, acidic pH or specific enzymatic activity—to maximize therapeutic efficacy at the target site [121]. Despite these design principles, significant challenges impede clinical translation. These include overcoming the physical and biological barriers of the bone microenvironment, improving targeting specificity and efficiency, ensuring carrier stability with low immunogenicity, enhancing drug penetration and retention within bone tissue and addressing scalability and reproducibility in manufacturing.

**Table 2:** Typical bone-targeting molecules.

Bone-targeting molecules	Structure	Advantages	Disadvantages	Reference
Bisphosphonate	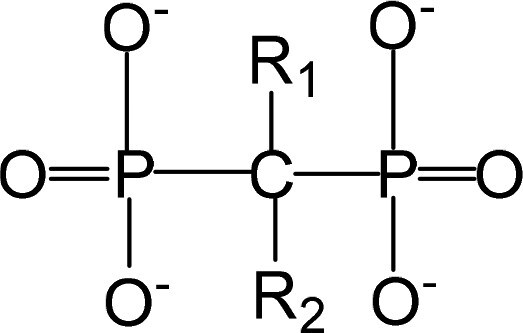	Enhance the affinity towards bone minerals Improve the pharmacological antiresorptive efficacy pharmacological activity Modulate the immune microenvironment of bone	Produce side effects Compromise the accessibility of the material Affect the activity of materials	[147–149]
Tetracycline	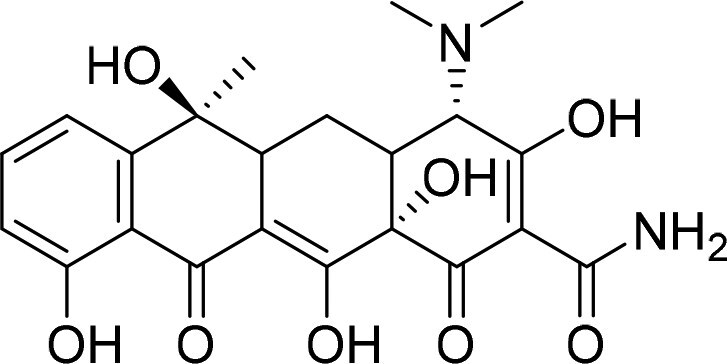	Improve bone-targeting ability and bone affinity Exhibit anti-inflammatory and antibacterial activities Enhance the biocompatibility of the material	Affect the emergence of bacterial resistance Affect the efficacy of the material Affect the optical properties	[111, 150]
Oligopeptide	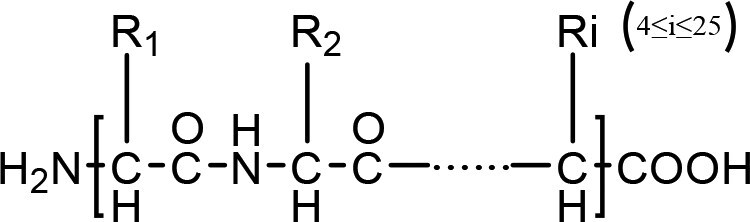	Easy to synthesize and functionalize Boost the bone affinity of the material Improve the biocompatibility of the material	Cause the functional instability of enzymes Restrict the permeability of biological tissues Result in complex characterization processes	[111]

### Development of polymer vesicles with bone-targeting effects for delivering antiresorptive drugs

In current research, various NP-based systems-both inorganic and organic-have been explored for targeted delivery of antiresorptive agents to bone injury sites. These include therapeutic peptides [151, 152] and nanomedicines [153–155] (such as silica NPs [156, 157], gold NPs [158, 159] and HAP NPs [160, 161]), which aim to modulate the osteoporotic microenvironment. Through strategic surface modification, these nanomaterials can achieve active bone targeting, thereby enhancing localized drug accumulation and improving therapeutic efficacy while minimizing systemic adverse effects.

**Figure 3:**
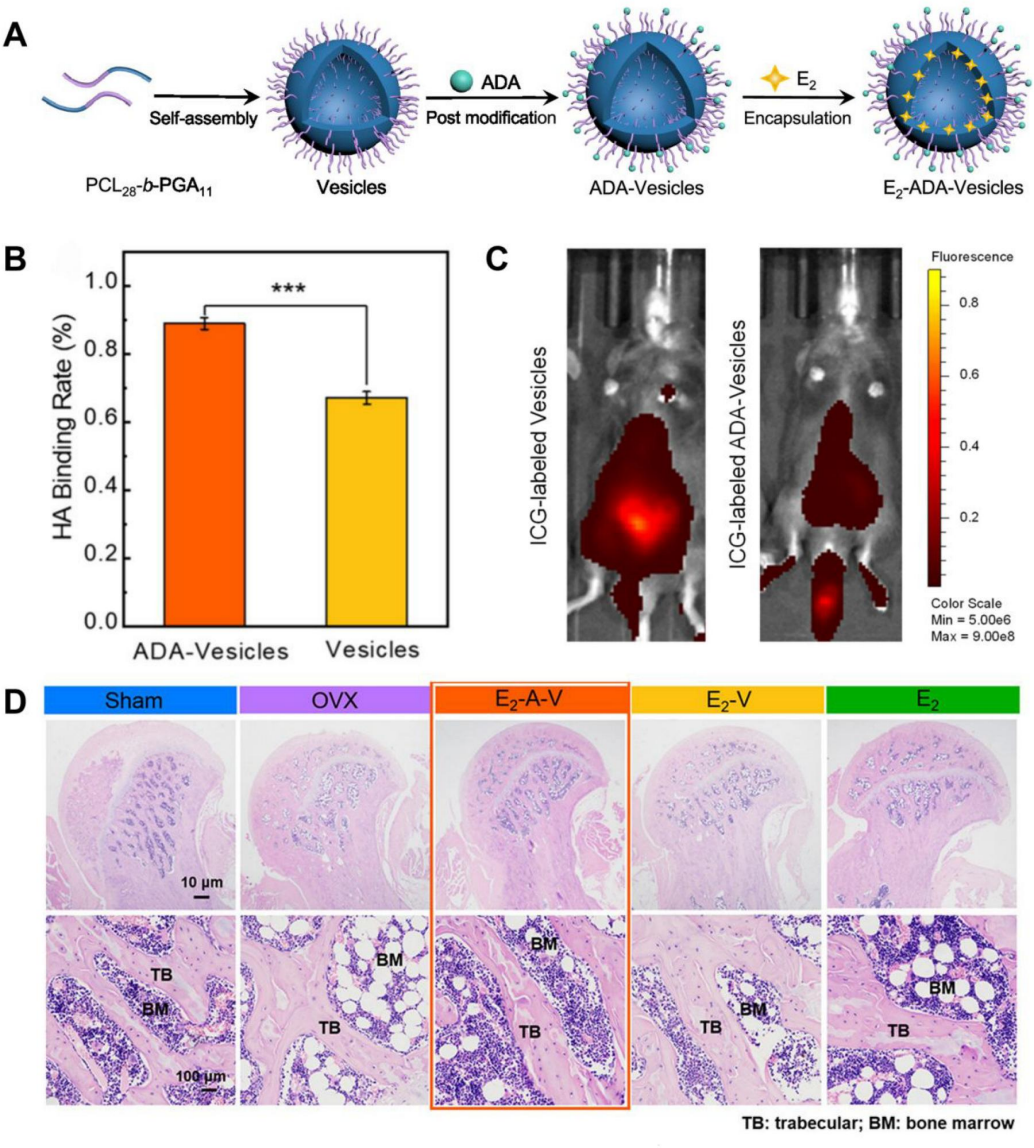
Polymeric nanovesicles with bone-targeting effects for delivering antiresorptive drugs. (**A**) Polymeric vesicles loaded with E_2_ synthesized by self-assembly of diblock copolymers. (**B**) HA binding capacity of vesicles and ADA-vesicles. (**C**) Biodistribution of ICG-labeled vesicles and ICG-labeled ADA vesicles 6 h after injection. (**D**) Pathologic observation of femur under different treatments. *P < 0.05, **P < 0.01 and ***P < 0.001. Bone-Targeting polymer vesicles for effective therapy of osteoporosis [162], Zhou *et al*., *Nano Letters*, 2021, by permission of Nano Letters. This image is not covered by the terms of the creative commons licence of this publication. For permission to reuse, please contact the rights holder.

Zhou *et al*. [162] designed a bone-targeted polymer vesicle for the effective treatment of PMOP. The polymeric vesicles were formed by self-assembly of PCL_28_-b-P[Glu_7_-stat-(Glu-ADA)_4_] block copolymers (Figure 3A). A biodegradable poly(ε-caprolactone) (PCL) membrane encapsulated E_2_, preventing premature release and ensuring protective effects during systemic circulation. Additionally, alendronic acid (ADA) is grafted onto the vesicle surface, conferring high affinity for bone tissue through HAP targeting. This modification enables selective E_2_ accumulation at sites of bone injury, maximizing local drug concentration while minimizing systemic exposure. Within the acidic microenvironment resulting from OC overactivation in osteoporotic bone, the vesicles undergo pH-responsive disintegration, leading to controlled release of E_2_ [163]. The locally released E_2_ subsequently promotes osteogenic activity. Furthermore, ADA is a potent bisphosphonate-synergistically contributes to the therapeutic outcome by suppressing OC activity and reducing bone resorption, thereby increasing BMD. Together, ADA-mediated targeting and antiresorptive effects, combined with E_2_-induced osteogenesis, result in a synergistic enhancement of bone regeneration and mineral density. Following the successful preparation of bone-targeted vesicles, it is essential to evaluate their bone-targeting capability. The *in vitro* bone affinity was assessed by measuring the binding capacity of both nontargeted vesicles and ADA-functionalized vesicles to HAP. As illustrated in Figure 3B, ADA-vesicles exhibited a significantly higher HAP binding rate compared to plain vesicles, indicating their enhanced ability to deliver E_2_ specifically to bone tissue. For *in vivo* evaluation, indocyanine green (ICG)-labeled polymer vesicles were administered via tail vein injection, and fluorescence imaging was performed. Figure 3C demonstrates that ADA-functionalized vesicles effectively accumulate within bone tissue, exhibiting superior bone-targeting efficiency compared to nontargeted vesicles. Pathological assessment after eight weeks of treatment revealed histological analysis indicating that, compared to the ovariectomized control group, bone tissue density and trabecular microarchitecture were preserved to varying degrees in the E_2_, E_2_-vesicle, and E_2_-ADA-vesicle groups. The protective effect was most pronounced in the E_2_-ADA-vesicle group (Figure 3D).

These results collectively confirm the outstanding bone repair efficacy of E_2_-ADA-vesicles, a bone-targeted polymeric vesicle system. This study ingeniously combines the osteogenic properties of E_2_ with the bone-targeting/antiresorptive characteristics of ADA, representing an advanced design in intelligent delivery systems.

### Reconstruction of the osteoporotic microenvironment using metal-organic framework materials

The high surface area and tunable porosity of nanomaterials enable efficient loading of anti-osteoporotic drugs or bioactive factors [164]. The porous architecture can further modulate drug release kinetics to achieve prolonged and controlled release, as well as influence cellular behavior-such as promoting M2 macrophage polarization-to ameliorate osteoporotic conditions [165, 166]. Metal-organic frameworks (MOFs) are a class of novel porous materials formed through the coordination-driven assembly of metal ions or clusters with multidentate organic ligands [167]. Within polymer encapsulation systems, the regulable nanochannels inherent to MOFs facilitate the precise design of unique polymeric components [168]. Furthermore, their prominent physicochemical properties—such as facile synthesis and functionalization, controllable pore size, structural diversity, high surface area and exceptional loading capacity—have enabled their widespread application across numerous fields [169].

**Figure 4:**
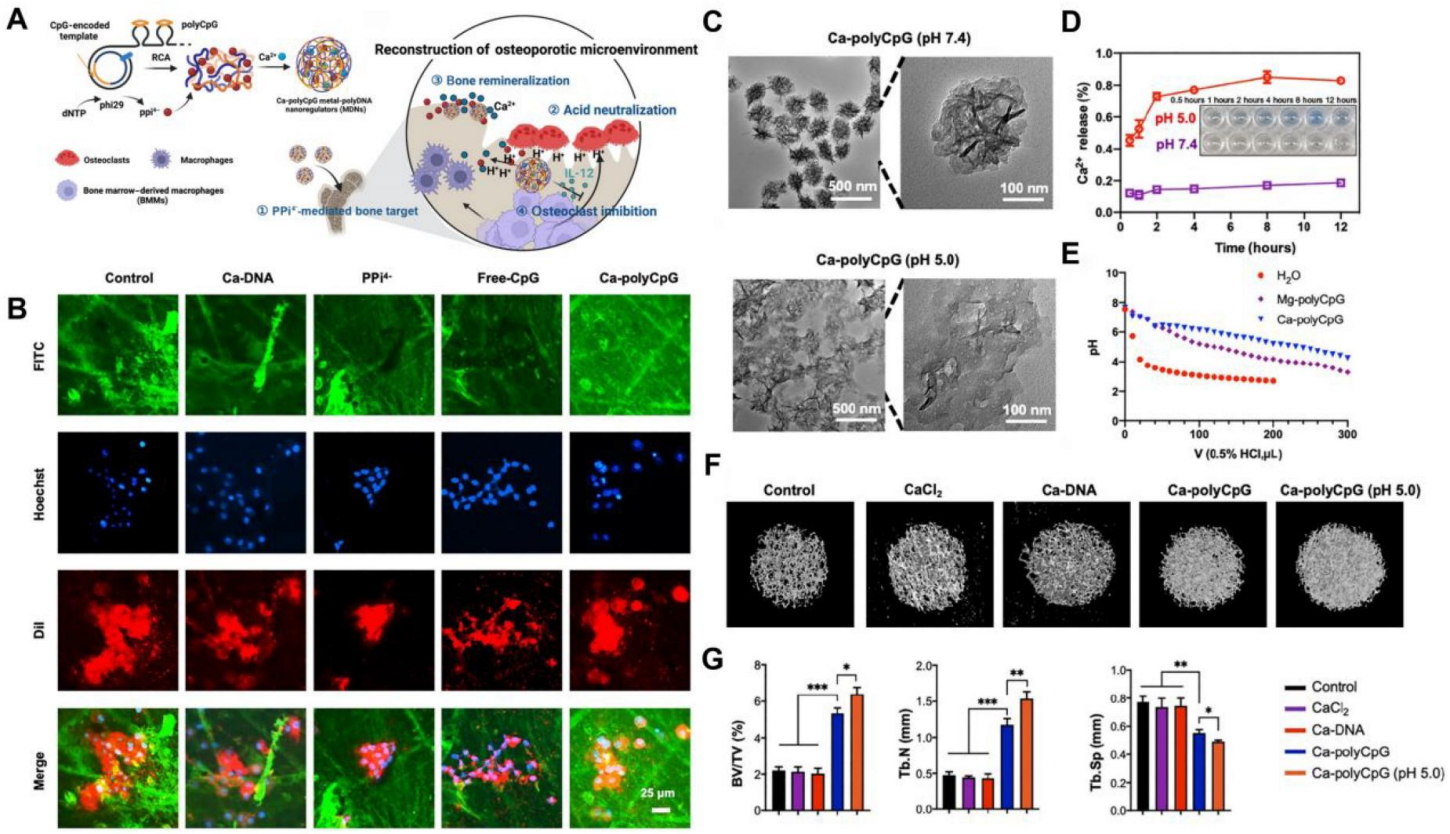
Reconstruction of the osteoporotic microenvironment using bioactive nanomaterials. (**A**) Schematic representation of Ca^2+^-polyCpG co-preparation for Ca-polyCpG MDNs. (**B**) Representative confocal images demonstrating extracellular acid resistance of Ca-polyCpG MDNs during osteoclast-mediated H^+^ excretion, measured via pH indicators. (**C**) TEM images of Ca-polyCpG MDNs incubated for 30 min at pH 5.0 and 7.4. (**D**) Ca^2+^ release from Ca^2+^-polyCpG MDNs following incubation at pH 5.0 and 7.4 for varying durations. (**E**) pH changes in Mg^2+^-polyCpG MDNs and Ca^2+^-polyCpG MDNs following acidification in hydrochloric acid titration experiments. (**F**) Representative three-dimensional micro-CT imaging of bone under different treatments. (**G**) Quantitative results from three-dimensional micro-CT analysis, expressed as BV/TV, Tb.N and Tb.Sp values. BV/TV: bone volume per tissue volume; Tb.N: trabecular number; Tb.Sp: trabecular separation. *P < 0.05, **P < 0.01 and ***P < 0.001. Metal-polyDNA nanoparticles reconstruct osteoporotic microenvironment for enhanced osteoporosis treatment [112]. Liu *et al*., *Science Advances*, 2023, by permission of Science Advances. This image is not covered by the terms of the creative commons licence of this publication. For permission to reuse, please contact the rights holder.

A significant limitation, however, arises from the use of small molecules as organic ligands, which often restricts functionality. To address this constraint, Liu *et al*. [112] proposed the substitution of conventional small-molecule ligands with biological macromolecules, specifically DNA and RNA. This approach enables the customization of multifunctional metal-DNA/RNA NPs via coordination with metal ions, as illustrated in Figure 4A. DNA and RNA molecules exhibit a high degree of specificity in binding to their target proteins, demonstrating superior molecular recognition capabilities. Furthermore, their intrinsic biodegradability enables rapid renal clearance *in vivo*, preventing long-term retention and associated bioaccumulation risks [170]. However, a significant challenge for metal-DNA/RNA NPs constructed from monovalent functional nucleic acids is their low stability in biological environments and poor cell membrane permeability [171]. To overcome these limitations, polynucleotide DNA (polyDNA) can be employed as a ligand. PolyDNA—an ultra-long single-stranded DNA (ssDNA) incorporating multivalent functional sequences—offers enhanced stability. By coordinating polyDNA with metal ions, it is possible to form multifunctional metal-polyDNA NPs (MDNs), a strategy that facilitates the design of diverse and bioactive nanomaterials. Multifunctional Ca-polyCpG MDNs effectively restore the osteoporotic microenvironment by neutralizing acidity, eliminating localized acidification caused by OC attachment to bone surfaces following depolarization. Furthermore, within the acidic osteoporotic microenvironment, they release Ca^2+^, thereby promoting bone tissue mineralization [172]. As depicted in Figure 4B, following OC labelling with DiI (1,1′-dioctadecyl-3,3,3′,3′-tetramethylindocarbocyanine perchlorate) and co-incubation with the pH probe fluorescein isothiocyanate (FITC), and subsequently observing fluorescence intensity on the bone surface to confirm local pH in osteoporotic bone during OC-mediated hydrogen ion excretion and the extracellular acid-buffering properties of Ca-polyCpG. It is evident that the green fluorescence signal is stronger in Ca-polyCpG MDN-treated samples, whereas excessive hydrogen ion secretion by OCs causes the FITC signal to nearly disappear. Thereby demonstrating Ca-polyCpG MDNs’ acid-neutralizing capacity. Transmission electron microscope (TEM) images reveal (Figure 4C) that Ca-polyCpG MDNs exhibit uniform structure at pH 7.4, yet undergo deformation under pH 5.5 conditions. Concurrently, the calcium colorimetric assay kit measured Ca^2+^ release from Ca-polyCpG MDNs at pH 7.4 and pH 5.0 (Figure 4D) revealing increased Ca^2+^ release under acidic conditions at pH 5.0. Concurrently, hydrochloric acid titration experiments demonstrated that both Ca-polyCpG MDNs and Mg-polyCpG MDNs significantly retarded pH decline (Figure 4E). To demonstrate that Ca^2+^ release promotes bone mineralization, micro-computed tomography (micro-CT) characterization was performed on osteoporotic bone tissue treated with Ca-polyCpG MDNs (Figure 4F and G), revealing a marked reversal of bone loss in the Ca-polyCpG MDNs group. This demonstrates the superior capacity of multifunctional Ca-polyCpG MDNs to enhance bone repair within the acidic microenvironment of OP.

In summary, the study by Liu *et al*. illustrates the considerable potential of nanotechnology to deliver coordinated interventions that target both skeletal and immune cells. This study employs an innovative strategy whereby MDNs neutralize the acidic microenvironment and release calcium ions, directly addressing the pathological characteristics of OP.

### Achieving balanced bone remodeling through synchronous regulation of osteogenesis and osteoclastogenesis using metal-based nanomaterials

Existing nanotechnology-based therapies for OP have demonstrated significant potential in systemic regulation, but still face several technical challenges. Most therapeutic strategies only focus on unidirectional regulation, such as promoting bone formation through osteoblastic differentiation or reducing bone resorption by inhibiting osteoclastic differentiation, rather than regulating both processes simultaneously. To address this, Chen *et al*. [173] developed multifunctional metal-phenolic networks (MPNs) NPs for systemic OP treatment, which can simultaneously regulate osteoblastic bone formation and osteoclastic bone resorption. MPNs are multifunctional nanomaterials formed by metal ions and polyphenolic compounds through coordination, which have shown potential application values in the biomedical fields in recent years, including the treatment of OP [174]. Each component in MPNs is involved in regulating bone metabolic balance: metal ions (Sr^2+^, Zn^2+^) can activate the Wnt/β-catenin pathway [175], enhancing OB activity and bone formation [176], polyphenolic compounds such as epigallocatechin gallate (EGCG) and TA possess antioxidant and anti-inflammatory properties, inhibiting the NF-κB signaling pathway and reducing bone marrow cell differentiation (by decreasing RANKL expression) [177]. Thus, MPNs based on metal ions can effectively regulate OB-mediated bone formation and OC-mediated bone resorption. Additionally, MPNs integrate the biological activities of metals and phenolic compounds, and can serve as pH-responsive carriers for drug delivery.

**Figure 5:**
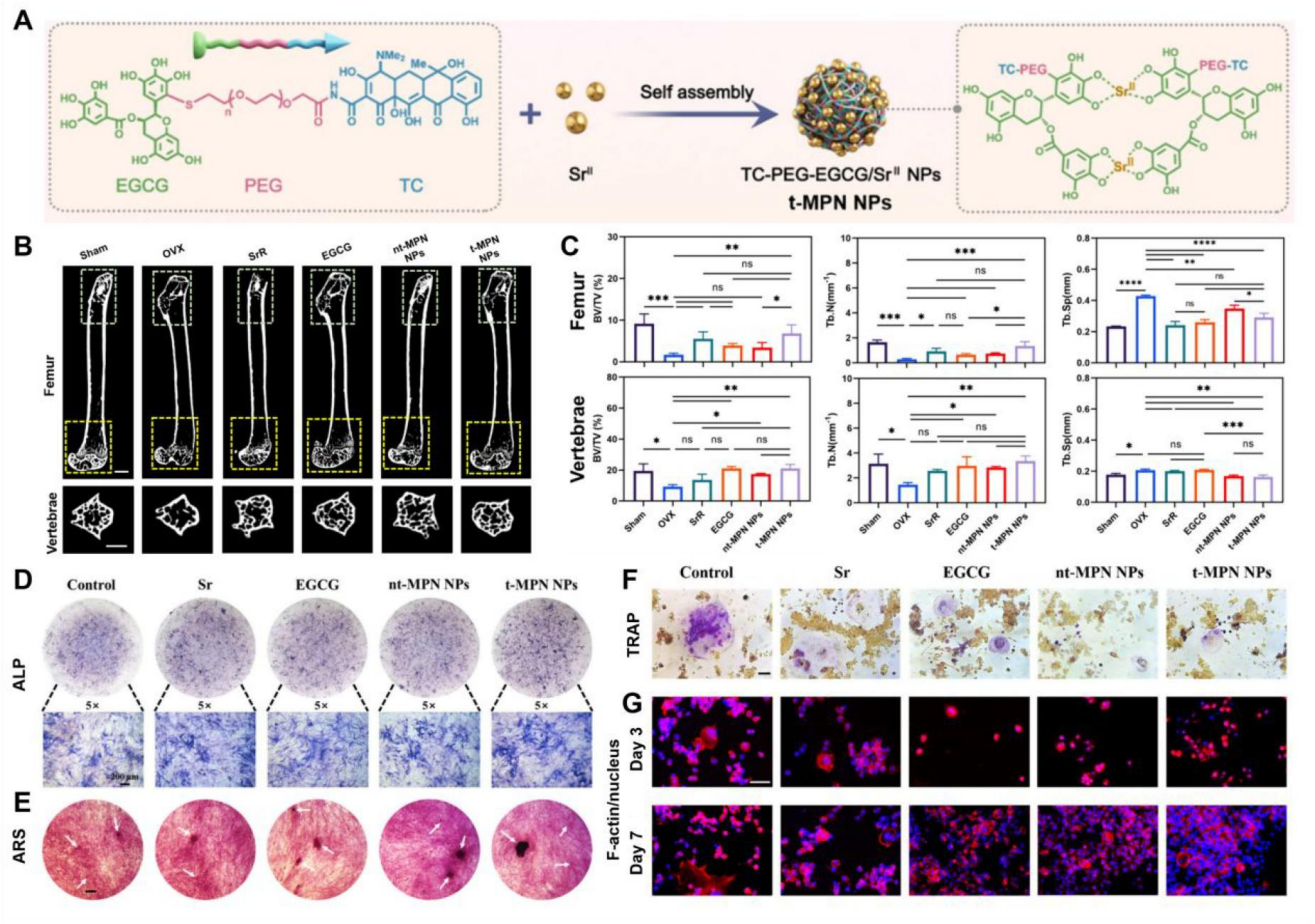
Remodeling bone balancing through synchronous regulation of osteogenesis and osteoclastogenesis. (**A**) Schematic representation of the synthesis of t-MPN NPs via EGCG-PEG-TC and Sr^2+^. (**B**) Representative micro-CT reconstructions of the femur and vertebrae in ovariectomized osteoporotic female mice treated with Sr, EGCG, nt-MPN NPs and t-MPN NPs. (**C**) Quantitative results of three-dimensional micro-CT analysis for femur and vertebrae under different treatments, expressed as BV/TV, Tb.N and Tb.Sp values. (**D**) ALP staining images of MC3T3-E1 cells at 7 days post-treatment. (**E**) MC3T3-E1 cell ARS staining images at Day 14 post-treatment. (**F**) TRAP staining images of RAW264.7 cells cultured for 7 days under different treatments. (**G**) F-actin and nucleus co-staining images of RAW264.7 cells at Day 3 and 7 post-treatment. *P < 0.05, **P < 0.01, ***P < 0.001, ****P < 0.0001, n.s. not significant. Regulation of bone remodeling by metal–phenolic networks for the treatment of systemic osteoporosis [173]. Chen *et al*., *ACS Applied Materials & Interfaces*, 2025, by permission of ACS Applied Materials & Interfaces. This image is not covered by the terms of the creative commons licence of this publication. For permission to reuse, please contact the rights holder.

The multifunctional MPN NPs consist of polyethylene glycol (PEG)-functionalized EGCG and Sr^2+^ (Figure 5A). Strontium (Sr) can simultaneously enhance osteogenesis and inhibit OC activity, with no obvious toxicity over a wide concentration range [178, 179]. Bone-targeted delivery can be achieved by grafting ALN onto PEG, which increases the accumulation of EGCG in bone sites in the internal circulation. Due to the excellent pH responsiveness of multifunctional MPNs, these NPs can release active components in the acidic microenvironment of cancellous bone, promote OB differentiation and reduce the number of activated OCs. As shown in Figure 5B and C, animal experiments were conducted to verify the *in vivo* therapeutic efficacy of MPN NPs. An ovariectomized mouse model was established and the mice were divided into groups: the Sham group served as the blank control, the OVX group as the negative control and the remaining groups were treated with Sr, EGCG, nt-MPN NPs and t-MPN NPs, respectively. After 4 weeks of treatment via tail vein injection in mice, the femurs and lumbar vertebrae were collected for analysis. Micro-CT images and quantitative analysis of diagnostic parameters for OP revealed that the key parameters of OVX mice treated with t-MPN NPs were almost restored to levels similar to those of normal mice, suggesting their potential in treating systemic OP. In addition, the ability of t-MPN NPs to simultaneously regulate OB-mediated bone formation and OC-mediated bone resorption was verified through cell interaction studies with MC3T3-E1 cells (a mouse preosteoblast cell line) and RAW264.7 cells (a mouse preosteoclast cell line). Under different treatment conditions, differentiation and mineralization assays were performed using ALP (Figure 5D) and alizarin red S staining (ARS) (Figure 5E), which showed that t-MPN NPs promoted OB differentiation. In OC experiments, both t-MPN NPs and nt-MPN NPs induced a smaller tartrate-resistant acid phosphatase (TRAP)-positive area in RAW264.7 cells, indicating inhibited OC differentiation (Figure 5F). Moreover, macrophages treated with t-MPN NPs exhibited the smallest cell area and the weakest fluorescence intensity of F-actin rings (Figure 5G), further verifying the inhibitory effect of t-MPN NPs on OC activation.

In summary, these findings confirm the feasibility of multifunctional MPN NPs in simultaneously regulating both OBs and OCs. By integrating Sr^2+^ and EGCG, MPN NPs achieve bidirectional regulation of osteogenesis and osteoclastogenesis, demonstrating the superior concept of synergistic therapy.

**Figure 6:**
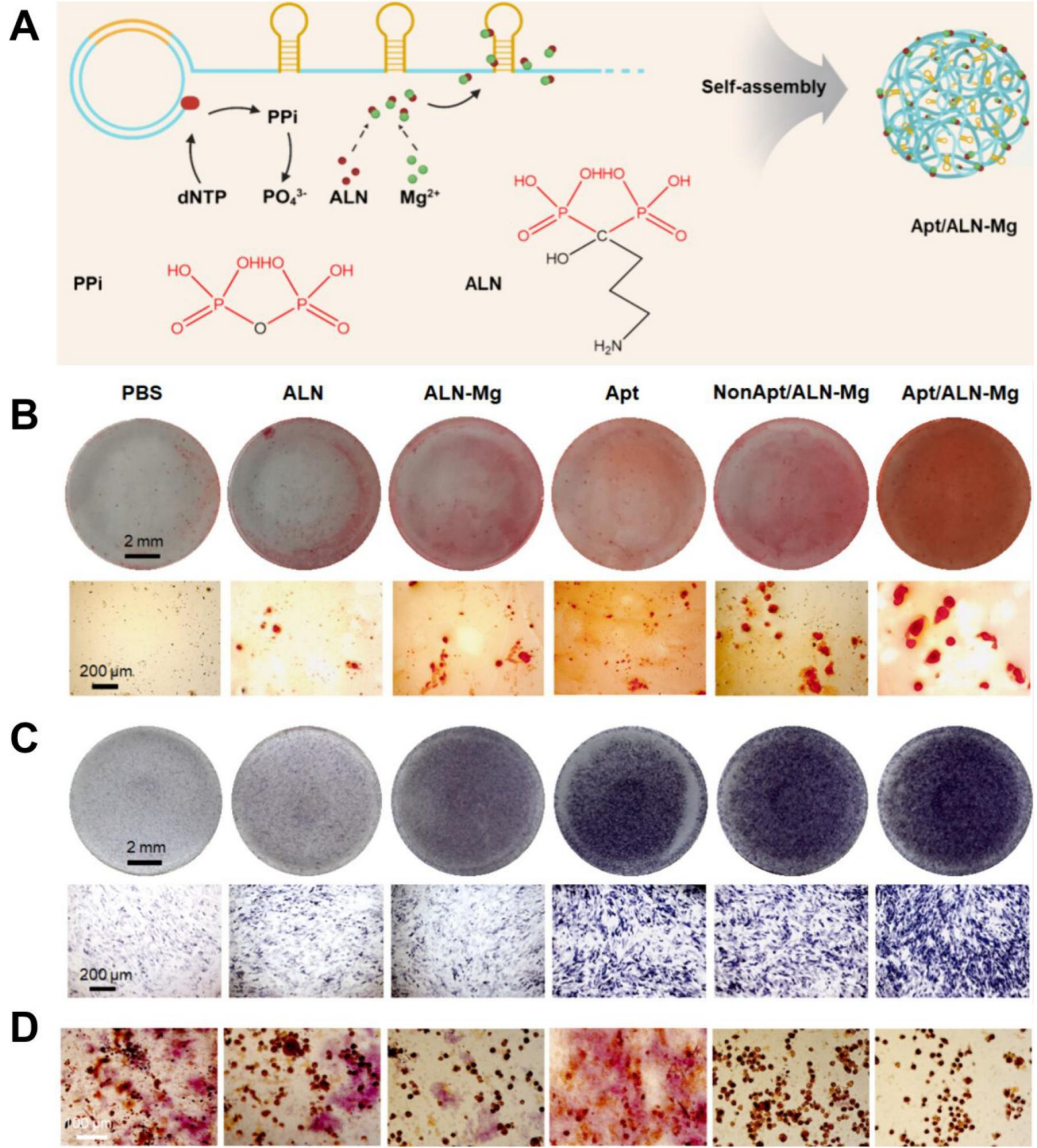
Sodium ALN nano-assemblies promote osteogenesis, inhibit osteoclast activity and combat OP. (**A**) Schematic diagram of the synthesis of Apt/ALN-Mg NPs. (**B**) ARS of MC3T3-E1 cells after 18 days of osteoblastic differentiation under different treatments. (**C**) ALP staining of rBMSCs after 7 days of osteoblastic differentiation under different treatments. (**D**) TRAP staining of RAW 264.7 cells on Day 5 after different treatments. Polyaptamer-driven crystallization of alendronate for synergistic osteoporosis treatment through osteoclastic inhibition and osteogenic promotion [84]. Yang *et al*., *ACS Nano*, 2025, by permission of ACS Nano. This image is not covered by the terms of the creative commons licence of this publication. For permission to reuse, please contact the rights holder.

Similarly, Yang *et al*. [84] also developed a nanoassembly that simultaneously inhibits bone resorption and promotes osteoblastic differentiation, and prepared a carrier-free dual-drug nanoassembly using the rolling circle amplification (RCA) reaction. DNA exhibits excellent biocompatibility and can drive crystallization through anisotropic nucleic acids, while Mg NPs show customizable biological functionality. In addition, DNA nanostructures have broad application prospects in the biomedical field due to their unique properties [180]. Sclerostin is an extracellular negative regulator of bone formation. It inhibits bone formation and promotes bone resorption by regulating related signaling pathways, and is a recognized therapeutic target for OP treatment [181, 182]. The antisclerostin DNA aptamer (Apt) is a single-stranded functional DNA structure that has high affinity for sclerostin and avoids the inherent limitations associated with antibodies, thus, facilitating progress in bone-specific OP treatment. Given the similarity between pyrophosphate (PPi) and bisphosphonates in their phosphate backbone structures, biomimetic co-assembly was achieved by modifying the RCA reaction: the aptamer targeting antisclerostin protein was co-assembled with the antiresorptive agent ALN to form Apt/ALN-Mg NPs (Figure 6A). To evaluate the effect on osteoblastic differentiation, rabbit bone marrow mesenchymal stem cells (rBMMSCs) and OBs (MC3T3-E1 cells) were treated with PBS, ALN, ALN-Mg, Apt, NonApt/ALN-Mg and Apt/ALN-Mg, respectively. Mineralized nodules (Figure 6B) and ALP (Figure 6C) in each group were stained. The staining images showed that rBMMSCs and MC3T3-E1 cells in the Apt/ALN-Mg group exhibited the highest ARS and ALP activities, respectively. Meanwhile, TRAP staining of RAW 264.7 macrophages (Figure 6D) revealed that multinucleated OCs were almost absent after treatment with Apt/ALN-Mg NPs or NonApt/ALN-Mg NPs, confirming the excellent OC-inhibiting efficacy of both Apt/ALN-Mg and NonApt/ALN-Mg NPs. In summary, Yang *et al*.’s Apt/ALN-Mg NPs represent a significant step forward in addressing DNA stability and reducing carrier toxicity through ingenious carrier-free design and metal coordination.

NPs based on metal-nucleic acid coordination structures offer significant advantages, yet they remain condition-dependent. First, while metal coordination enhances structural integrity, the DNA scaffold remains susceptible to degradation by serum nucleases, potentially leading to structural disassembly and loss of function. Second, nucleic acid molecules themselves possess immunostimulatory properties; particularly DNA containing specific sequences (such as unmethylated CpG motifs) can activate innate immune responses via Toll-like receptor 9 (TLR9), triggering the release of inflammatory cytokines. Finally, achieving efficient bone-targeted systemic delivery requires overcoming a series of cascading obstacles. Following injection, NPs are immediately enveloped by blood components, forming a “protein coat.” This layer of proteins completely obscures their surface-modified targeting molecules, alters their size, surface charge and *in vivo* behavior, potentially leading to failure of bone targeting and rapid clearance by the mononuclear phagocytic system (e.g. liver and spleen) [183]. Bone exhibits relatively low vascularization [184], requiring NPs to extravasate from blood vessels and traverse the interstitial to reach the bone surface. Future research should focus on enhancing NPs stability, optimizing their immunocompatibility and comprehensively evaluating their bone tissue distribution efficiency using large animal OP models.

### Bone-targeting liposomes indirectly modulate bone remodeling processes by activating the organ-skeleton axis to stimulate the secretion of biological signals

Previous studies have demonstrated that bone-targeted nanomedicines diffuse to nontargeted organs during circulation, such as accumulating in the liver [185, 186]. Even highly effective targeted drugs are not exclusively localized to bone tissue. A fraction of the administered dose invariably enters the systemic circulation and is subject to metabolism and clearance by other organs [187, 188]. The liver and spleen constitute the primary clearance organs for NPs, where resident macrophages (Kupffer cells in the liver [189, 190] and splenic macrophages [191]) recognize and phagocytose these foreign entities [192]. Provided the nanomedicine demonstrates high biocompatibility and degradability, this accumulation is typically transient and reversible, posing a low risk of significant acute toxicity. Nonetheless, chronic or high-dose administration regimens warrant careful evaluation of potential risks, such as chronic inflammation or organ damage. Regarding renal clearance, NPs with an extremely small hydrodynamic diameter (<5–6 nm) may be eliminated via glomerular filtration [193]. Larger particles risk renal retention, necessitating thorough safety assessments of glomerular and tubular function to preclude nephrotoxicity.

**Figure 7:**
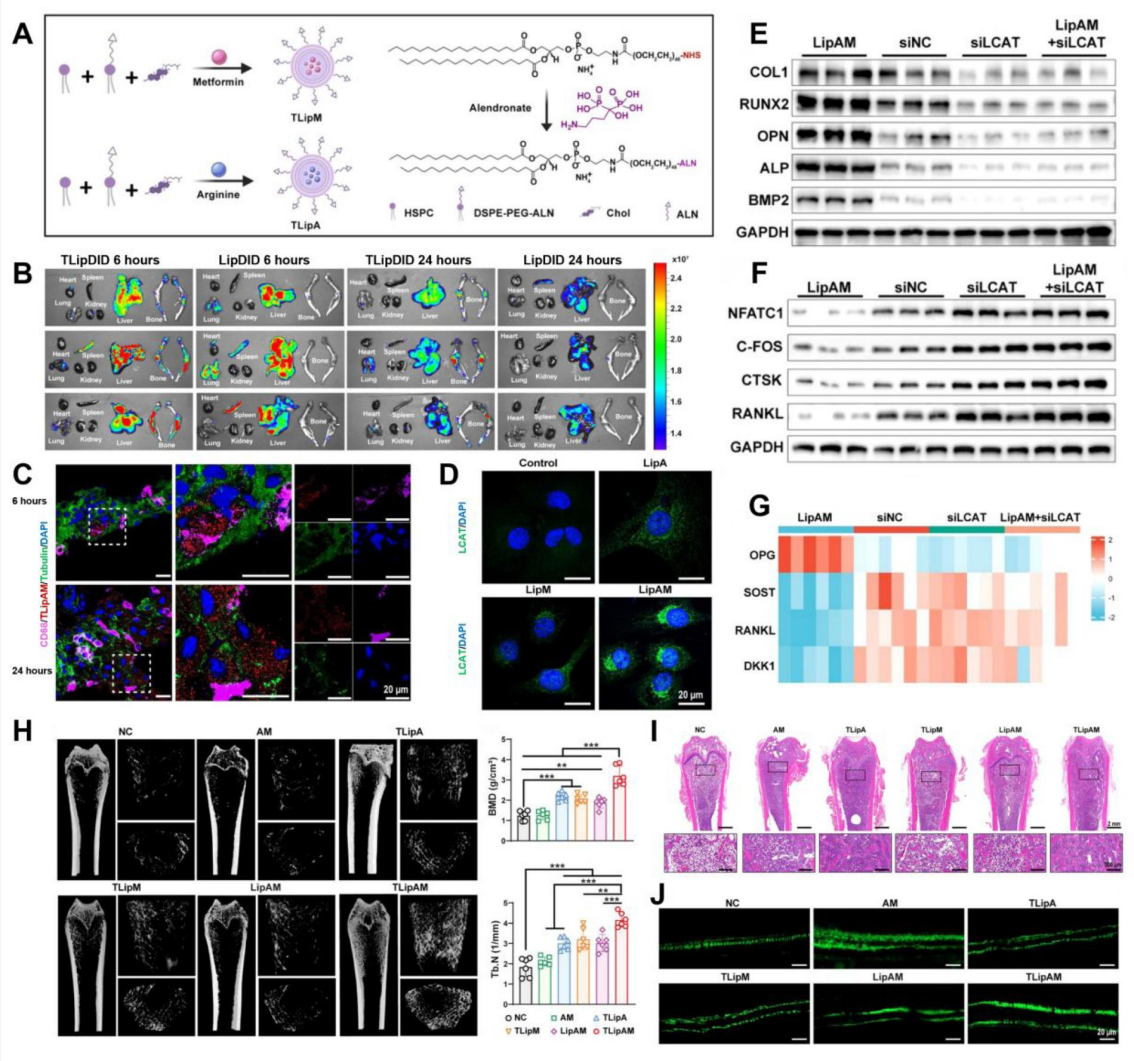
Bone-targeted liposome-encapsulated antiresorptive agents for improving OP. (**A**) Schematic diagram of the synthesis of liposomes TLipM and TLipA. (**B**) Biodistribution in organs and bone tissue at 6 h and 24 h post-injection of LipDID and TLipDID. (**C**) Immunofluorescence staining images of liver tissue at 6 h and 24 h post-injection of TLipAM. Scale bar: 20 μm. (**D**) Immunofluorescence staining images of LCAT in hepatocytes under different treatments. Scale bar: 20 μm. (**E**) Protein expression levels of COL1, RUNX2, OPN, ALP and BMP2 in BMMSCs cultured for 5 days under different HCM conditions. (**F**) Protein expression levels of RANKL, NFATC1, CTSK and CFOS in RAW 264.7 cells cultured for 6 days under different HCM conditions. (**G**) ELISA assay of OPG, SOST, RANKL and DKK1 in MLO-Y4 cells following different treatments. (**H**) Quantitative analysis of micro-CT images and BMD versus Tb.N in the distal femur under different treatments. (**I**) Representative H&E-stained images of the distal femur under different treatments. (**J**) Dynamic calcein-fluorescein dual-labelled trabecular images. *P < 0.05, **P < 0.01, ***P < 0.001. Integration of Bone-Targeted delivery and crosstalk modulation of Liver-Bone axis for improved osteoporosis therapy [194]. You *et al*., *ACS Nano*, 2025, by permission of ACS Nano. This image is not covered by the terms of the creative commons licence of this publication. For permission to reuse, please contact the rights holder.

Such distribution patterns result in reduced concentrations of nanomedicines within skeletal tissues, prompting speculation regarding their potential biological effects. Consequently, You *et al*. [194] developed a novel bone-targeting liposomal system co-encapsulating arginine and metformin for the treatment of OP. Surprisingly, beyond their direct effects on bone cells (OBs, OCs and osteocytes), these liposomes were found to stimulate hepatic secretion of LCAT. This liver-derived hormone has been shown to inhibit OC maturation and potentiate OB activity [195, 196]. The selection of cargo was based on a multiorgan targeting rationale. Arginine, a conditionally essential amino acid, not only promotes osteogenic differentiation [197] but also facilitates liver regeneration and repair [198], thereby demonstrating dual regulatory capabilities in bone and liver homeostasis. Metformin, a classic hypoglycemic agent, exerts pleiotropic effects beyond glycemic control, including the positive regulation of osteogenesis [199]. It enhances the expression of bone morphogenetic protein-2 (BMP-2) and the phosphorylation of its downstream effectors Smad1/5/9, synergizing with the AMPK pathway to promote bone formation [200, 201]. To achieve targeted delivery, the researchers engineered liposomes to co-encapsulate both drugs and functionalized the liposomal surface with the bone-homing molecule ALN, thereby enhancing specific drug accumulation at skeletal sites (Figure 7A).

To validate the bone-targeting efficacy and assess the *in vivo* biodistribution of the liposomes, both targeted (functionalized) and nontargeted liposomes were labeled with the near-infrared fluorescent dye 1,1′-dioctadecyl-3,3,3′,3′-tetramethylindocarbocyanine, 4-chlorobenzenesulfonate salt (DiD). In vivo imaging revealed that from 6 to 24 h post-injection, the fluorescence intensity in both bone and liver regions gradually decreased (Figure 7B). This attenuation indicates that while initial accumulation occurred in both targeted and nontargeted organs, the liposomes demonstrated a favorable clearance profile. Further analysis via fluorescence microscopy of liver sections showed that the combined liposomal formulation (LipAM) initially accumulated primarily around Kupffer cells within 6 h. By 24 h, however, a substantial redistribution of the fluorescent signal was observed throughout the hepatic parenchyma (Figure 7C), confirming the ability of the liposomes to subsequently enter hepatocytes. Consistent with this hepatic uptake, analysis of LCAT expression in hepatocytes demonstrated that treatment with LipAM induced a more potent upregulation of LCAT compared to other groups (Figure 7D), supporting its enhanced efficacy in stimulating the secretion of this osteogenic hormone. To elucidate the mechanism through which LipAM stimulates hepatic LCAT secretion and subsequently promotes osteogenesis, conditioned medium (LipAM HCM) was collected from hepatocytes treated with LipAM and applied to cultures of OBs, OCs and osteocytes. The protein expression of key osteogenic and osteoclastogenic markers were analyzed via Western blotting, respectively. The results indicated that LipAM HCM significantly enhanced the expression of osteogenic markers at protein levels (Figure 7E), while concurrently suppressing the expression of proteins associated with osteoclastogenesis (Figure 7F). Enzyme-linked immunosorbent assay (ELISA) further corroborated that LipAM HCM promoted osteogenic differentiation of OBs while inhibiting OC differentiation (Figure 7G). Subsequent *in vivo* studies validated the therapeutic efficacy of LipAM, identifying it as the most effective treatment for post-traumatic OP among the tested groups (Figure 7H–J). Comprehensive analyses, including micro-CT reconstruction, hematoxylin and eosin (H&E) staining and calcein double-labeling, collectively demonstrated that LipAM treatment significantly enhances bone repair and regeneration. You *et al*. proposed a bone-targeted “liver-bone axis” synergistic therapy, achieving a paradigm shift in treatment philosophy from “single-site therapy” to “multiorgan system regulation.” This study breaks through the traditional framework of “bone-local” treatment by activating the liver-bone axis (LCAT hormone) to achieve systemic bone protection, offering a novel perspective.

### Nanomedicines targeting energy metabolism alleviate cellular senescence and reverse bone loss by maintaining mitochondrial homeostasis

MSCs are multipotent stromal cells capable of differentiating into various cell lineages. Under the influence of specific physicochemical cues and signaling pathways, they can be directed to undergo osteogenic differentiation. Furthermore, MSCs contribute to bone homeostasis by secreting a variety of paracrine factors that modulate the bone microenvironment, thereby facilitating bone regeneration and repair. Consequently, the senescence of MSCs is associated with a decline in their functional capacity, impairing bone and cartilage regeneration and contributing to the pathogenesis of age-related musculoskeletal disorders, such as OP. At the cellular level, senescence is frequently accompanied by mitochondrial dysfunction, characterized by a reduction in mitophagy activity and the subsequent accumulation of damaged mitochondria. Furthermore, given the central role of mitochondria in cellular metabolism, their dysfunction is itself a potent driver of MSCs senescence. A key mechanism underlying this energy failure is the impairment of mitochondrial ATP synthase. This critical enzyme utilizes the proton gradient across the inner mitochondrial membrane to synthesize ATP, the primary energy currency that powers the vast majority of cellular activities [202]. Emerging evidence suggests that ATP synthase plays a crucial role in ameliorating mitochondrial dysfunction through its regulatory functions, thereby attenuating senescence in MSCs [203].

**Figure 8:**
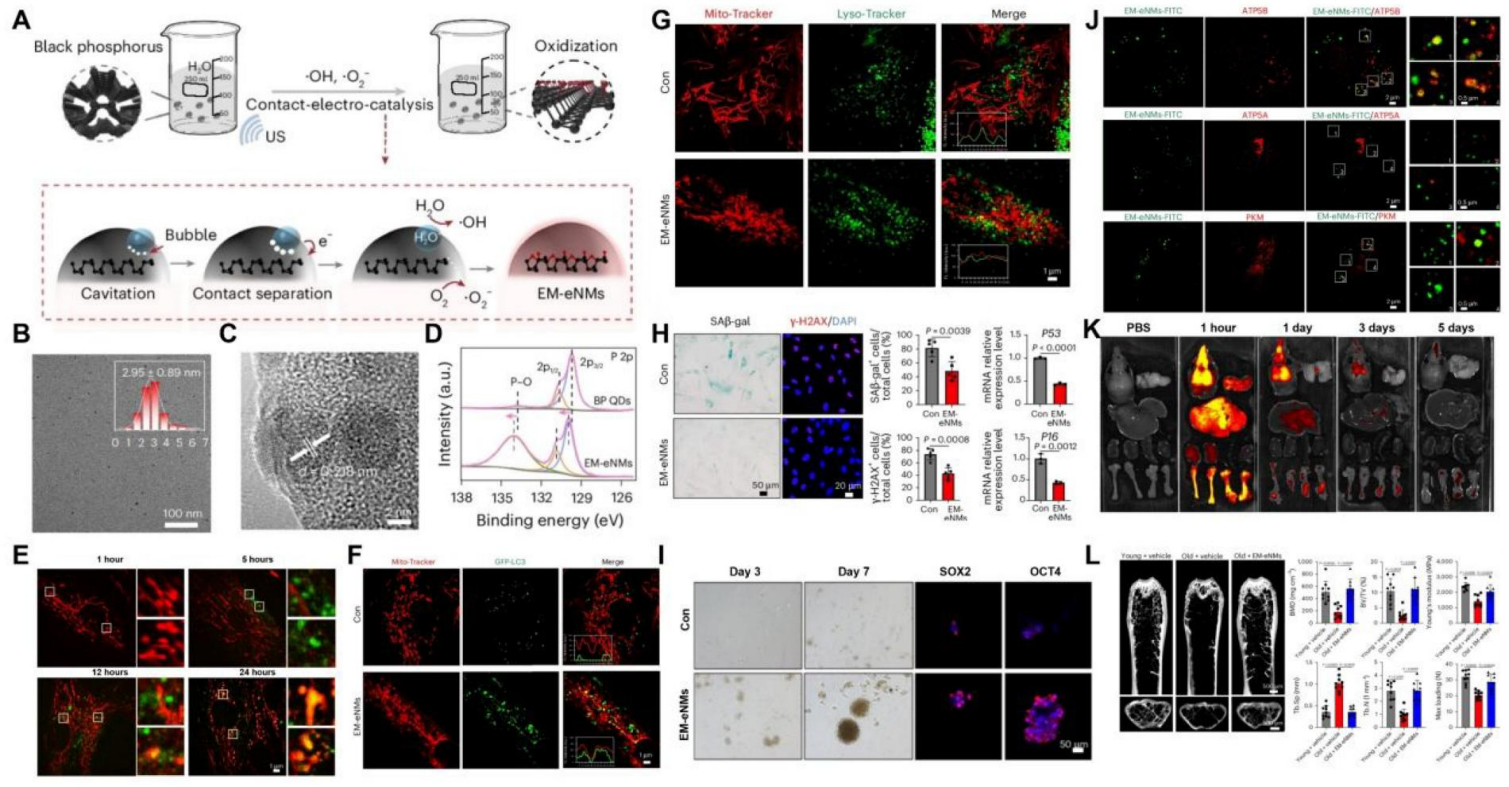
Nanodrugs involved in energy metabolism reshape the bone microenvironment by maintaining mitochondrial homeostasis and alleviating cellular senescence. (**A**) Schematic illustration of EM-eNMs preparation via contact electrocatalysis. (**B**) Representative TEM image of EM-eNMs. (**C**) Representative HRTEM image of EM-eNMs. (**D**) XPS spectra of BP QDs and EM-eNMs. (**E**) Immunofluorescence imaging revealing intracellular localization of EM-eNMs at different time points. (**F**) Super-resolution microscopy demonstrating increased mitochondrial autophagosome formation following GFP-LC3 adenovirus transduction of BMMSCs. (**G**) Co-staining with Mito-Tracker (left) and Lyso-Tracker (middle) to label mitochondria and lysosomes respectively. (**H**) β-galactosidase staining and immunofluorescence staining of γ-H2AX in PBS- and EM-eNMs-treated BMMSCs, along with semi-quantitative analysis. (**I**) Microscopic images and semi-quantitative analysis of PBS- and EM-eNMs-treated BMMSCs at Day 3 and 7. (**J**) Immunofluorescence imaging of BMMSCs treated with EM-eNMs-FITC for 24 h and stained for ATP5Β, ATP5Α or PKM. (**K**) IVIS imaging of EM-eNMs-FITC biodistribution. (**L**) Representative micro-CT images and semi-quantitative analysis of trabecular bone parameters. An energy metabolism-engaged nanomedicine maintains mitochondrial homeostasis to alleviate cellular ageing [204]. Chen *et al*., *Nature Nanotechnology*, 2025, by permission of Nature Nanotechnology. This image is not covered by the terms of the creative commons licence of this publication. For permission to reuse, please contact the rights holder.

Chen *et al*. [204] developed energy metabolism-targeting nanomedicines (EM-eNMs) that mitigate bone loss by regulating mitochondrial homeostasis in BMMSCs, thereby restoring their stem cell properties and pluripotency. Leveraging the advantageous properties of nano-interfaces, the researchers synthesized these nanomedicines using ultra-small black phosphorus quantum dots (BP QDs) as precursors. Through a process of contact electrocatalysis, the BP QDs surfaces were selectively oxidized to generate EM-eNMs featuring highly exposed surface phosphate groups (Figure 8A). TEM analysis of the synthesized EM-eNMs revealed an average diameter and surface morphology (Figure 8B) consistent with those of pristine BP QDs [205], indicating that the surface oxidation process did not alter the core NP size. High-resolution TEM (HRTEM) imaging (Figure 8C) identified a lattice fringe spacing of 0.218 nm, which corresponds to the (002) crystal plane of BP, thereby confirming the preservation of the quantum dot crystalline structure. Furthermore, X-ray photoelectron spectroscopy (XPS) analysis (Figure 8D) confirmed the successful formation of a highly exposed phosphate surface structure, as evidenced by the presence of phosphorus species in a highly oxidized state.

*In vitro* cellular experiments investigated the subcellular localization of EM-eNMs within aged BMMSCs. FITC-labelled NPs revealed the highest fluorescence intensity in the mitochondrial region after 24 h, indicating EM-eNMs were internalized and accumulated within mitochondria (Figure 8E). Autophagy activity was assessed by monitoring LC3 protein dynamics via adenovirus-mediated GFP-LC3 expression. The EM-eNMs-treated group exhibited a significant increase in the co-localization area between GFP-LC3 puncta and mitochondria (Figure 8F), suggesting an enhancement in mitophagosome formation. The intracellular localization and distribution of the mitochondrial chaperone protein Hsp60 and the lysosomal marker LAMP1 were assessed via immunofluorescence co-staining. As shown in Figure 8G, an increased colocalization signal between Hsp60 and LAMP1 was observed following EM-eNMs treatment, indicating promoted mitochondria-lysosome contact formation. This result confirms the enhancement of late-stage mitophagy. Collectively, these findings demonstrate that EM-eNMs facilitate mitochondrial fission and autophagic clearance, mechanisms which may contribute to the restoration of intracellular homeostasis and the attenuation of senescence in BMMSCs. To evaluate long-term induction effects, an *in vitro* cellular experimental protocol was similarly employed: human BMMSCs were continuously treated with EM-eNMs from the fourth passage (P4) until the tenth to twelfth passages (P10-12), after which their biological characteristics were assessed. The anti-aging efficacy of EM-eNMs was confirmed by a significant reduction in the number of SA-β-gal- and γ-H2AX-positive cells, as quantified from representative images (Figure 8H). This was further supported by qPCR analysis, which showed suppressed expression of the aging-related genes P53 and P16. Spheroid formation assays under low-adhesion conditions revealed that EM-eNMs-treated cells exhibited elevated expression of the pluripotency markers SOX2 and OCT4, as demonstrated by immunofluorescence (Figure 8I), indicating a maintenance of stem cell characteristics in aged BMMSCs. It is noteworthy that EM-eNMs, as mitochondrial-targeted nanomedicines, function not merely as passive drug carriers but as active “mimetic regulators” of cellular energy metabolism. Their mechanism centers on the direct intervention of mitochondrial ATP synthase, the core engine of OXPHOS. During ATP production, the catalytic β-subunit (ATP5B) of this synthase is critical for driving the energy-dependent processes that sustain cellular function. To experimentally validate ATP5B as the primary target of EM-eNMs within the mitochondrial matrix, immunofluorescence co-localization analysis was performed. As shown in Figure 8J, the signal for ATP5B exhibited the most pronounced overlap with those of ATP synthase F1 subunit (ATP5A) and pyruvate kinase (PKM), confirming the specific localization of EM-eNMs at this key catalytic site and their role in reprogramming cellular metabolism to counteract senescence.

Additionally, *in vivo* and *ex vivo* experiments were conducted on aged mice. Following intravenous injection of FITC-labelled EM-eNMs, *in vitro* analysis revealed that after systemic administration, EM-eNMs selectively accumulated in bone tissue (Figure 8K). Histomorphometry analysis of femoral sections (Figure 8L) confirmed that EM-eNM treatment enhanced BMD, increased mineralization deposition rates and improved trabecular number. Collectively, these findings demonstrate their potential for preventing and treating age-related OP. This study reverses mesenchymal stem cell senescence by targeting mitochondrial energy metabolism, providing a fundamental therapeutic strategy for age-related OP.

While nanotechnology targeting mitochondria holds considerable therapeutic potential, it must be squarely acknowledged that mitochondria are highly dynamic and sensitive hubs of energy production and cellular signaling. The introduction of exogenous nanomaterials entails inherent risks that warrant careful scrutiny. Specifically, off-target interactions with other membrane-bound organelles—such as the endoplasmic reticulum or lysosomes—cannot be fully excluded, which may interfere with essential physiological functions. Furthermore, excessive or prolonged induction of mitochondrial fission and autophagy risks perturbing the delicate balance of cellular energy metabolism. Given that mitochondrial network dynamics are pivotal in determining cell fate, pushing “clearance and regeneration” beyond physiological limits could impair ATP synthesis and promote the accumulation of metabolic intermediates, thereby aggravating cellular stress. Future advances in this field will, therefore, depend on the development of more intelligent and precisely controllable nanosystems, informed by a deeper understanding of mitochondrial biology in bone cells.

As previously mentioned, the primary bottleneck in the clinical translation of nanotechnology for systemic drug delivery via intravenous injection in OP treatment lies in the body’s complex biological barriers and long-term safety concerns. Upon entering the body, nanomaterials are recognized as foreign substances by liver and spleen macrophages, leading to their phagocytosis and clearance. This results in significant off-target drug accumulation, reduces bone-targeting efficiency, and may cause hepatosplenic toxicity [206]. The glomerular filtration barrier acts like a “precise sieve,” efficiently allowing only rigid, negatively charged or neutral NPs with hydrated diameters below approximately 6–8 nm to pass through [207]. Particles exceeding this threshold cannot be cleared via urine, constituting the primary cause of long-term accumulation. Research confirms that designing ultra-small nanomedicines with diameters of 3–4 nm enables highly efficient renal targeting and clearance [208]. Additionally, NPs as exogenous substances may nonspecifically activate the immune system, triggering acute/chronic inflammation, cytokine storms or immunosuppression. Studies indicate that IL-12-loaded PLGA nanospheres can alter pharmacokinetics [206], converting systemic immune activation into localized tissue inflammation, thereby significantly reducing immunotoxicity while maintaining therapeutic efficacy. In summary, nanotechnology presents revolutionary opportunities for targeted OP therapy, yet systemic administration safety remains a complex systems engineering challenge involving materials, biology and immunology. Future breakthroughs hinge on developing next-generation nanomedicines that are “precisely targeted, intelligently released and ultimately safely cleared.” This requires deep integration and collaborative innovation across materials science, pharmacology, toxicology and clinical medicine.

## Conclusion and outlook

Over the past few decades, the integration of nanotechnology has addressed critical limitations associated with conventional OP treatments, facilitating a shift from broad-spectrum systemic drug administration toward precision-targeted therapeutic strategies. The rational design of advanced nanocarriers has enabled breakthroughs in overcoming longstanding challenges such as poor bioavailability, nonspecific biodistribution and limited mechanistic diversity [209, 210]. By leveraging bone-targeting nanomaterials, these innovative delivery systems enhance the directed transport of pharmaceuticals to skeletal tissues, showing pronounced potential in the management of OP. Surface functionalization with specific ligands further improves drug solubility and stability, reduces systemic toxicity and increases overall bioavailability. More importantly, such modifications facilitate active targeting, allowing nanocarriers to precisely recognize and bind to bone cells at pathological sites, thereby significantly minimizing off-target effects and improving therapeutic efficacy [211, 212]. Additionally, the nanoplatform can co-deliver multiple drugs with distinct functions, simultaneously regulating osteoclastic and osteoblastic processes to achieve synergistic effects, thereby fundamentally promoting bone homeostasis. Building upon these advantages, research into drug delivery systems based on bone-targeting nanomaterials has yielded numerous breakthroughs.

**Figure 9:**
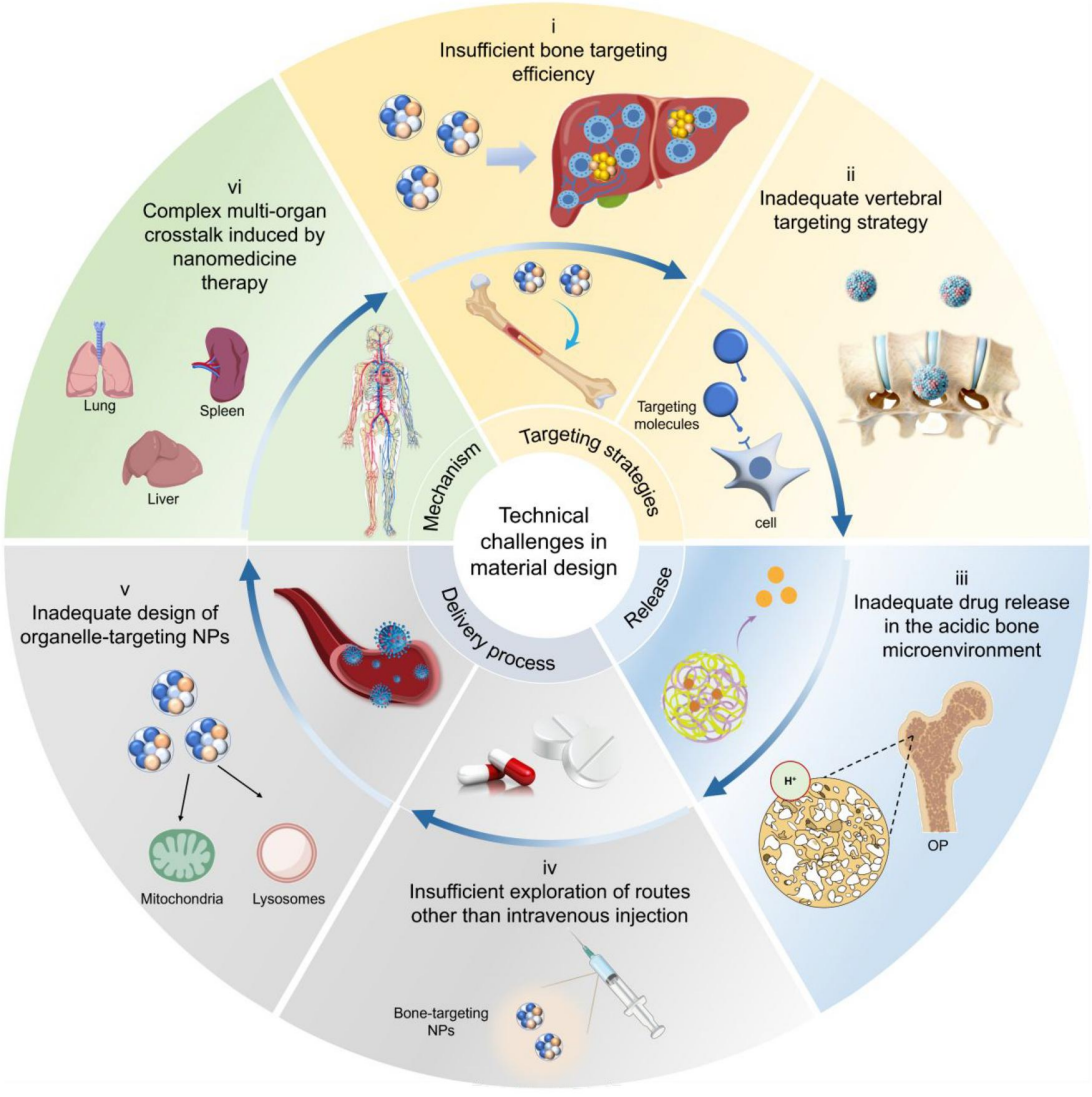
Material design challenges for bone-targeting nanomaterials in OP treatment.

However, bone-targeted nanomedicines still face numerous challenges in terms of technological optimization (Figure 9): (i) Limitations in target specificity and enrichment efficiency. Although nanocarriers can passively target bone tissue, a portion of the drug is still sequestered by the mononuclear phagocyte system in organs such as the liver and spleen [213], resulting in suboptimal bone-targeting efficiency. Moreover, existing targeting strategies primarily rely on bone matrix affinity, making it difficult to precisely distinguish between OCs and OBs, thereby compromising therapeutic specificity. (ii) Current bone-targeting NPs encounter difficulties in vertebral targeting. Within the osteoporotic microenvironment, the metabolic activity of vertebral trabecular bone differs from that of the femur, potentially impairing the release efficiency of pH-responsive NPs. Moreover, existing bone-targeting strategies exhibit significantly lower enrichment rates in vertebrae compared to the femur. (iii) Difficulties in controlling drug release persist, as the pH of the osteoporotic bone microenvironment differs little from that of normal bone. This results in slow or incomplete drug release from pH-responsive NPs, failing to achieve the objective of sustained-release therapy. (iv) Current research on bone-targeting nanomaterials for OP treatment primarily focuses on intravenous administration, with challenges remaining for delivery routes such as oral or intraperitoneal injection. (v) The challenge of penetrating biological barriers: for nanomedicines to act upon bone marrow MSCs and OBs, they must effectively traverse multiple biological barriers including bone tissue, vascular walls and cell membranes. Ultimately, they must also enter cells and target specific organelles. Many nanomedicines struggle to effectively reach and enter target cells to exert their effects due to unsuitable size, charge or surface chemistry. Consequently, designing nanosystems capable of efficiently traversing these barriers represents a significant challenge. (vi) Complexity of mechanism studies and individual variability: Nanodrugs not only act upon bone cells but can also indirectly influence bone metabolism by modulating hepatic secretion of LCAT. This indicates that the *in vivo* mechanisms of nanodrugs may be far more complex than designed, involving multiorgan crosstalk. Consequently, individual variations such as patient age, underlying causes of OP and hepatic function status may significantly impact nanodrug metabolism and ultimate efficacy.

Despite demonstrating significant potential in preclinical studies, bone-targeting nanomaterials face multiple challenges in clinical translation. The primary issue is insufficient assessment of long-term safety and biocompatibility. The interaction between nanomaterials and biological systems is unique and complex, necessitating systematic evaluation of their long-term *in vivo* fate and potential toxicity. For instance [214], while bisphosphonate-modified nanocarriers enhance bone targeting, high doses of bisphosphonates themselves may induce adverse reactions such as osteonecrosis of the jaw and nephrotoxicity. The biodegradation rates and metabolic pathways of inorganic nanomaterials (e.g. HAP, calcium carbonate) remain incompletely understood, necessitating further investigation into risks associated with long-term retention, including immune responses and tissue fibrosis. Second, quality control challenges in large-scale production pose significant hurdles. Consistency in batch manufacturing of nanomedicines remains a critical technical bottleneck for clinical translation. Bone-targeting nanosystems often feature complex structures involving the precise assembly of multiple functional components. Factors such as the surface modification density of targeting ligands, the integration method of responsive elements and the uniformity of drug encapsulation all decisively influence the efficacy and safety of the final product. Finally, the issue of regulatory assessment arises, with the complexity of characterizing nanomedicines being the primary concern: The critical quality attributes of nanomedicines encompass not only chemical composition and purity but also physical parameters such as particle size distribution, surface charge, morphology and aggregation state, alongside biological characteristics like protein coating composition and cellular uptake efficiency. This multidimensional characterization necessitates an interdisciplinary approach to integration, while the lack of standardized testing protocols further complicates regulatory evaluation.

In summary, despite the aforementioned challenges, drug delivery systems based on bone-targeting nanomaterials remain an ideal choice for treating bone disorders. This offers optimized solutions for enhancing drug targeting efficiency and refining drug release mechanisms. On the one hand, developing multilevel targeting strategies involves further optimizing the design of targeting ligands. This entails selecting molecules with higher bone affinity or employing multiligand synergistic targeting to enhance efficiency, thereby achieving cellular targeting. This is accomplished by conjugating antibodies or peptides capable of specifically recognizing surface markers of OBs or BMMSCs onto NPs surfaces. Concurrently, flexible application of diverse nanomaterials should prioritize biodegradable options, offering viable strategies for oral and intraperitoneal administration in adjuvant therapies. This ensures safe *in vivo* degradation and clearance of NPs. Future research necessitates further clinical trials to validate the therapeutic potential of these novel formulations.

## Supplementary Material

rbag065_Supplementary_Data
