# Supplementary material for: Advanced bone-targeted nanomaterials for the systemic treatment of osteoporosis
Source: Regen Biomater. 2026 Apr 1;13:rbag065. doi: 10.1093/rb/rbag065 (PMC13186200; doi:10.1093/rb/rbag065)
Supplement: rbag065_Supplementary_Data [file rbag065_supplementary_data.docx]

**Table 1.** A list of abbreviations.

| Osteoporosis | OP | Bone mineral density | BMD | Postmenopausal osteoporosis | PMOP | Senile osteoporosis | SOP |
| --- | --- | --- | --- | --- | --- | --- | --- |
| Osteoclasts | OCs | Hydroxyapatite | HAP | Osteoblasts | OBs | Mesenchymal stem cells | MSCs |
| Alkaline phosphatase | ALP | Osteocalcin | OCN | Reactive oxygen species | ROS | Receptor activator of nuclear factor κB ligand | RANKL |
| Interleukin | IL | Tumor necrosis factor α | TNF-α | Nanoparticles | NPs | Octylcolchicine | OI |
| Senescence-associated secretory phenotype | SASP | Matrix metalloproteinases | MMPs | Bone marrow mesenchymal stem cells | BMMSCs | Bone morphogenetic protein 9 | BMP9 |
| Insulin-like growth factor 1 | IGF-1 | Lecithin cholesterol acyltransferase | LCAT | Transforming growth factor | TGF | Short-chain fatty acids | SCFAs |
| Lipopolysaccharides | LPS | Prostaglandin E2 | PGE2 | Selective estrogen receptor modulators | SERMs | Parathyroid hormone | PTH |
| Self-assembled peptide nanofibers | SAPNs | MicroRNA | miRNA | Nitric oxide | NO | Extracellular vesicles | EVs |
| Estradiol | E_2_ | Alendronic acid | ADA | Indocyanine green | ICG | Alendronate | ALN |
| Metal-organic frameworks | MOFs | Polynucleotide DNA | polyDNA | Single-stranded DNA | ssDNA | Metal-polyDNA NPs | MDNs |
| Micro-computed tomography | Micro-CT | Toll-like receptor 9 | TLR9 | Metal-phenolic networks | MPNs | Strontium | Sr |
| Alizarin red S staining | ARS | Rolling circle amplification | RCA | Rabbit bone marrow mesenchymal stem cells | rBMMSCs | Transmission electron microscope | TEM |
| X-ray photoelectron spectroscopy | XPS | Epigallocatechin gallate | EGCG | Hematopoietic stem cell | HSC | Osteopontin | OPN |
| Oxidative phosphorylation | OXPHOS | Adenosine triphosphate | ATP | Fibroblast growth factor | FGF | Thioketal | TK |
| Polyethylene glycol | PEG | Fluorescein isothiocyanate | FITC | Tartrate-resistant acid phosphatase | TRAP | Tannic acid | TA |
